# Investigating laryngeal squamous cell carcinoma: clinical features and the role of *TAS2R16* polymorphisms and its protein levels

**DOI:** 10.1007/s12672-025-02898-1

**Published:** 2025-06-12

**Authors:** Enrika Pileckaite, Alvita Vilkeviciute, Vykintas Liutkevicius, Rasa Liutkeviciene

**Affiliations:** 1 Neuroscience Institute, Medical academy, Lithuanian University of Health Sciences, Kaunas, LT-44307 Lithuania; 2 Department of Otorhinolaryngology, Lithuanian University of Health Sciences, Kaunas, LT-44307 Lithuania

## Abstract

**Background:**

Laryngeal squamous cell carcinoma (LSCC) is the second most common malignancy of the respiratory tract after lung cancer, presents symptoms like hoarseness, sore throat, and dysphagia, and about 150,000 new cases are diagnosed worldwide annually. Risk factors such as tobacco smoking, alcohol consumption, and genetic variations, including *TAS2R16* polymorphisms, significantly influence LSCC development. Recent research suggests TAS2R16, a bitter taste receptor, may play a role in inflammation regulation and could be linked to cancer susceptibility, particularly in individuals with alcohol and nicotine dependency.

**Methods:**

A total of 312 LSCC patients and 320 healthy controls participated in the study. Deoxyribonucleic acid (DNA) was extracted using salting-out technology. Real time polymerase chain reaction was used for genotyping. Using the ELISA technique, serum levels were measured.

**Results:**

The distribution of TT, CT, and CC genotypes of *TAS2R16* rs860170 is statistically significantly different in groups: LSCC patients, both early-stage and late-stage LSCC patients, patients without metastasis and control group. Results showed that *TAS2R16* rs1357949 GG and AG genotypes together are associated with decreased odds of developing LSCC in non-smoking patients under the dominant model. Also, each rs1357949 G allele was found to decrease the odds of LSCC occurrence in non-smokers under the additive model. TAS2R16 serum levels in the LSCC were greater in *TAS2R16* rs978739 CT genotype carriers than in the control group.

**Conclusions:**

The distribution of *TAS2R16* rs860170 genotypes varies notably between LSCC patients, including those at early and late stages, as well as those without metastasis. Additionally, rs1357949 GG and AG genotypes show a protective effect against LSCC development in non-smokers, with the G allele reducing the odds of occurrence. Higher serum levels of TAS2R16 were observed in LSCC patients with the rs978739 CT genotype, suggesting a potential link between these genetic variations and LSCC pathophysiology.

## Introduction

Head and neck squamous cell carcinoma is the seventh most common cancer in the world, of which about 25% consists of laryngeal squamous cell carcinoma (LSCC) [1]. LSCC is the second most common malignancy of the respiratory tract tumor, after lung cancer [2]. Symptoms of the LSCC include hoarseness, sore throat, dysphagia, painful swallowing, otalgia, cough and expectoration of blood [3]. About 150,000 new LSCC cases are diagnosed worldwide every year [1]. The overall survival rate has not significantly improved despite continuous improvements in the diagnosis and treatment of laryngeal cancer because of the tumor’s enigmatic occurrence and high recurrence rate [4]. According to the European Cancer Information System 190 new cases of laryngeal cancer were identified in Lithuania in 2022, most of them were diagnosed in men (178 cases) [5]. In recent years, LSC) has shown novel epidemiological traits. The incidence rate of laryngeal cancer has increased among young people, especially those under 40 years old. This tendency may be brought on by variables including changing patterns of alcohol and tobacco use, rising HPV infection rates, and bad lifestyle choices [4]. The disease is characterized by a different clinical course, prognosis and treatment, depending on the site of onset in the larynx [2]. The most favorable clinical course of LSCC was determined for the tumor that appeared in the vocal cords, due to the weak lymph flow and rarely detected metastases in regional neck lymph nodes [6]. Based on data from the Surveillance, Epidemiology, and End Results Program, the five-year relative survival rate for cancer that develops in the vocal cords is approximately 77% [7]. While the laryngeal supraglottis and subglottis regions have a rich lymphatic network, cancer in these areas is more likely to result in metastases to the regional lymph nodes [6]. The occurrence of LSCC is influenced by risk factors [8]. According to the American Cancer Society, risk factors such as gastroesophageal reflux, Plummer-Vinson syndrome, genetic inheritance, exposure to chemicals, asbestos, nickel or ionizing radiation, and some viral infections are associated with the development of laryngeal cancer [9]. Also, it has been proven that tobacco smoking and alcohol consumption play an important role in the carcinogenesis of this tumor [10]. Inflammation signaling pathways and xenobiotic metabolizing enzymes may contribute to carcinogenesis and malignant transformation by regulating the cell cycle, cell survival, angiogenesis, and invasiveness. Genetic variations, such as single nucleotide polymorphisms (SNPs), can significantly impact carcinogenesis by altering the normal function of proteins encoded by genes involved in these cellular mechanisms [11]. Bitter taste receptors (TAS2Rs) play a significant role in human health, and the factors determining their ligand specificity remain largely unclear [12]. TAS2Rs are part of the G-protein-coupled receptor family, were first discovered in taste buds, and act as peripheral receptors for bitter stimuli. Recent research has revealed that TAS2Rs are also expressed in various extra-gustatory tissues, such as the respiratory tract, gastrointestinal mucosa, urethra, heart, and gingiva. Activation of TAS2Rs can inhibit the production of inflammatory mediators induced by lipopolysaccharide (LPS) in both human whole blood and lung macrophages, indicating a potential role for TAS2Rs in regulating inflammation tightly [13]. TAS2R16 is one of the best-studied bitter taste receptors at the molecular and population level [14]. The receptor-mediated response to beta-glucopyranosides is mediated by the gene of *TAS2R16*, which is part of the TAS2R family [15]. *TAS2R16* is not so widely studied, most polymorphisms of this gene are used in population studies, determining the change of protein functions or the effects of evolution [16, 17]. Numerous research has been carried out to investigate the role of taste receptor genes in innate immunity, ranging from cell culture tests to human and animal studies [15]. Several single nucleotide polymorphisms (SNPs) of the *TAS2R16* are considered risk factors for alcohol dependence [12] or associated with longevity [18]. There are at least 17 polymorphisms in human *TAS2R16* alone, that are related to alcohol dependency [12]. Additionally, *TAS2R16* genetic variations have been linked to African Americans developing nicotine dependency [19]. We aim to investigate the influence of *TAS2R16* gene polymorphisms on the occurrence of LSCC, as these variants are associated with alcohol and nicotine dependence – significant risk factors for the development of this disease. Finding more effective targets for both prediction and therapy is therefore urgently needed. However, data examining the relationships between *TAS2R16* polymorphisms and the pure group of LSCC patients, as well as the effect on the survival rate of patients, are still lacking. By analyzing *TAS2R16* gene SNPs, we seek to understand their potential role in predisposing individuals to LSCC, given the established link between alcohol consumption and increased cancer risk and also evaluate the five years survival rates.

## Materials and methods

### Study design and structure

From 2009 to 2024, this case-control research was carried out at the Lithuanian University of Health Sciences (LUHS), Kaunas, Lithuania, at the Department of Otorhinolaryngology and LUHS, Kaunas, Lithuania, Neuroscience institute, Laboratory of Ophthalmology. The Kaunas Regional Biomedical Research Ethics Committee gave its approval to the research protocol (BE-2-37), date of issue: 25 March 2019. Every method used in the study complied with the Declaration of Helsinki and its later revisions, the institution’s ethical guidelines, or comparable ethical standards. Every participant in the research gave their informed permission.

Our study involved 632 individuals divided into control (*n* = 320) and patients with LSCC (*n* = 312) groups. The LSCC patient group consisted of 312 men with an average age of 61.3 years. The control group consisted of 320 men with an average age of 61.6 years. When comparing the groups of subjects according to age, we did not find statistically significant differences (*p* = 0.081). However, during the study, we collected information about the lifestyle habits of individuals. We gathered data from 173 LSCC patients and 119 healthy individuals. In the LSCC group, smokers and alcohol drinkers accounted for 87.9% of each group, while in the healthy group, smokers consisted of 18.5% and alcohol drinkers – 49.7%. Demographic data of the subjects are presented in Table 1.

**Table 1:** Demographic data

Characteristics	LSCC group, *n* = 312	Control group, *n* = 320	*p*-value
Age median (IQR)	62 (10)	64 (9)	0.081*
Smoking habits, *n* (%) Smokers Non-smokers			
	152 (87.9)	22 (18.5)	
	21 (22.1)	97 (81.5)	
Alcohol consumption, *n* (%) Drinkers Non-drinkers			
	152 (87.9)	58 (49.7)	
	21 (22.1)	61 (51.3)	
Stage, *n* (%) I II III IV			
	109 (34.9)		
	66 (21.2)		
	53 (17.0)		
	84 (26.9)		
Tumor size (T), *n* (%) 1 2 3 4			
	113 (36.2)		
	66 (21.2)		
	59 (18.9)		
	74 (23.7)		
Metastasis to the neck lymph nodes (N), *n* (%) 0 1 2 3			
	250 (80.1)		
	19 (6.1)		
	40 (12.8)		
	3 (1.0)		
Distant metastasis (M), *n* (%) 0 1			
	308 (98.7)		
	4 (1.3)		
Tumor cell differentiation grade (G), *n* (%) 1 2 3			
	89 (28.5)		
	198 (63.5)		
	25 (8)		

LSCC – laryngeal squamous cell carcinoma; *p*-value – significance level and Bonferroni corrected significance level when *p* = 0.05/3

#### Control group

Patients who were consulted in the Department of Otorhinolaryngology, LUHS, and scheduled for surgical treatment (tympanoplasty, ossiculoplasty, tympanostomy, nasal bone reposition septoplasty, rhinoseptoplasty, uvulopalatopharyngoplasty, or radiofrequency thermoablation of the soft palate) were enrolled into the present study. Additionally, the inclusion criteria for the control group: those above the age of 18; signed a document requesting informed consent. The exclusion criteria for the control group: chronic infectious and non-infectious diseases (diabetes mellitus, malignant tumors, systemic connective tissue disorders, hypertension, coronary artery disease, stroke, or problems after organ or tissue transplantation are examples of systemic illnesses); use of sedatives or antiepileptic medications; subjects younger than 18 years of age.

#### LSCC group

The inclusion criteria for the LSCC group: those above the age of 18; pathologist-confirmed diagnosis of LSCC. All patients with suspected LSCC underwent a thorough otorhinolaryngological examination at the Department of Otorhinolaryngology’s Outpatient Office, which included flexible endoscopy and/or video laryngostroboscopy. A biopsy and direct micro laryngoscopy were performed on each patient. The LUHS Department of Pathology verified the histopathological diagnosis of LSCC. The final LSCC diagnosis with staging was established by doing neck and laryngeal computed tomography (CT) scans or magnetic resonance imaging (MRIs). The National Comprehensive Cancer Network (NCCN)-accepted Guidelines for Head and Neck Cancers Classification, Version 2.2020, were used in the staging of LSCC [20]. The exclusion criteria for the LSCC group: those with another type and location of cancer; those with acute or chronic infectious diseases; those taking psychomotor suppressants and antiepileptic medications; those under the age of 18. However, The Lithuanian State Register of Death Cases and Their Causes, Institute of Hygiene, Vilnius, provided the LSCC group statistics on the death rate, including the survival period following LSCC diagnosis.

### Sample collection and DNA extraction

Peripheral venous blood samples from these patients were collected in ethylenediaminetetraacetic acid (EDTA) containing vacutainer tubes for DNA extraction and vacutainer tubes without any anticoagulants for protein measurements. The tube with EDTA was stored in a freezer at − 80 °C until extraction of the DNA, while the tubes without any anticoagulants were kept for 30 min at room temperature, in a vertical position, then centrifuged for 10 min with 1900 Relative Centrifugal Force (RCF). Following that, the vacuum tube’s blood serum was aspirated into a sterile Eppendorf tube and kept in a freezer at -80 °C until it was needed for the study.

DNA extraction was carried out in the Neuroscience Institute’s Ophthalmology Laboratory at the Lithuanian University of Health Sciences. The DNA salting-out technique was used to extract DNA samples from venous blood. The salting-out method was performed according to previous study [21].

### Genotyping

*TAS2R16* rs860170, rs978739, rs1357949 SNPs were identified using TaqMan^®^ genotyping assays (Thermofisher Scientific, Pleasanton, CA, USA). A Step One Plus real-time polymerase chain reaction (RT-PCR) system (Applied Biosystems, Foster City, CA, USA) was used to genotype *TAS2R16* rs860170, rs978739, and rs1357949 in accordance with the manufacturer’s instructions. The RT-PCR was conducted using the Allelic discrimination program. The program used the fluorescence intensity of the several detectors (VIC and FAM) to determine the specific genotypes of each SNP. To verify the same rate of genotypes from the initial and recurrent genotyping, 5% of randomly chosen samples were genotyped for each of the SNPs.

### Protein concentration measurement

Twenty-three LSCC patients and twenty control subjects had their serum TAS2R16 levels measured. A commercially available enzyme-linked immunosorbent assay (ELISA) kit for human TAS2R16 (Abbexa LTD; Cambridge, UK) was used to measure the protein TAS2R16 concentration in LSCC patients. Following the manufacturer’s instructions, a microplate reader (Multiskan FC microplate photometer, Thermo Scientific, Waltham, MA, USA) was used to measure optical density right away at a wavelength of 450 nm. The standard curve, which has a sensitivity of < 0.1 ng/mL and a sensibility range of 0.312 to 20 ng/mL, was used to compute the TAS2R16 level.

### Statistical analysis

Statistical analysis was performed with IBM SPSS Statistics 29.0.1.0 software (IBM Corp, Armonk, NY, USA). Age of the patients and protein TAS2R16 concentration were presented as median with interquartile range (IQR). Using the Mann-Whitney U test, data that were not normally distributed between the two groups or subgroups were compared. The distribution of *TAS2R16* rs860170, rs978739, and rs1357949 was compared between the LSCC groups and the control group using the chi square (χ2) test. To assess the impact of genotypes on the onset of LSCC, we also conducted binary logistic regression analysis, yielding odds ratios (OR) and 95% confidence intervals (CI). The Akaike information criterion (AIC) was used to choose the most suitable genetic model. The AIC states that the best inheritance model is the one with the lowest value. Since we looked at three SNPs in the *TAS2R16* gene, we changed our significance threshold for multiple comparisons to alpha = 0.017 (0.05/3), and we defined statistically significant changes as those with *p* < 0.05. The Life-Table approach was used to analyze the survival rates of LSCC patients. Gehan’s criteria was used to compare survival rates across various genotypes of selected SNPs.

## Results

### Influence of *TAS2R16* rs860170, rs978739, rs1357949 SNPs on the development of LSCC

**Fig. 1:**
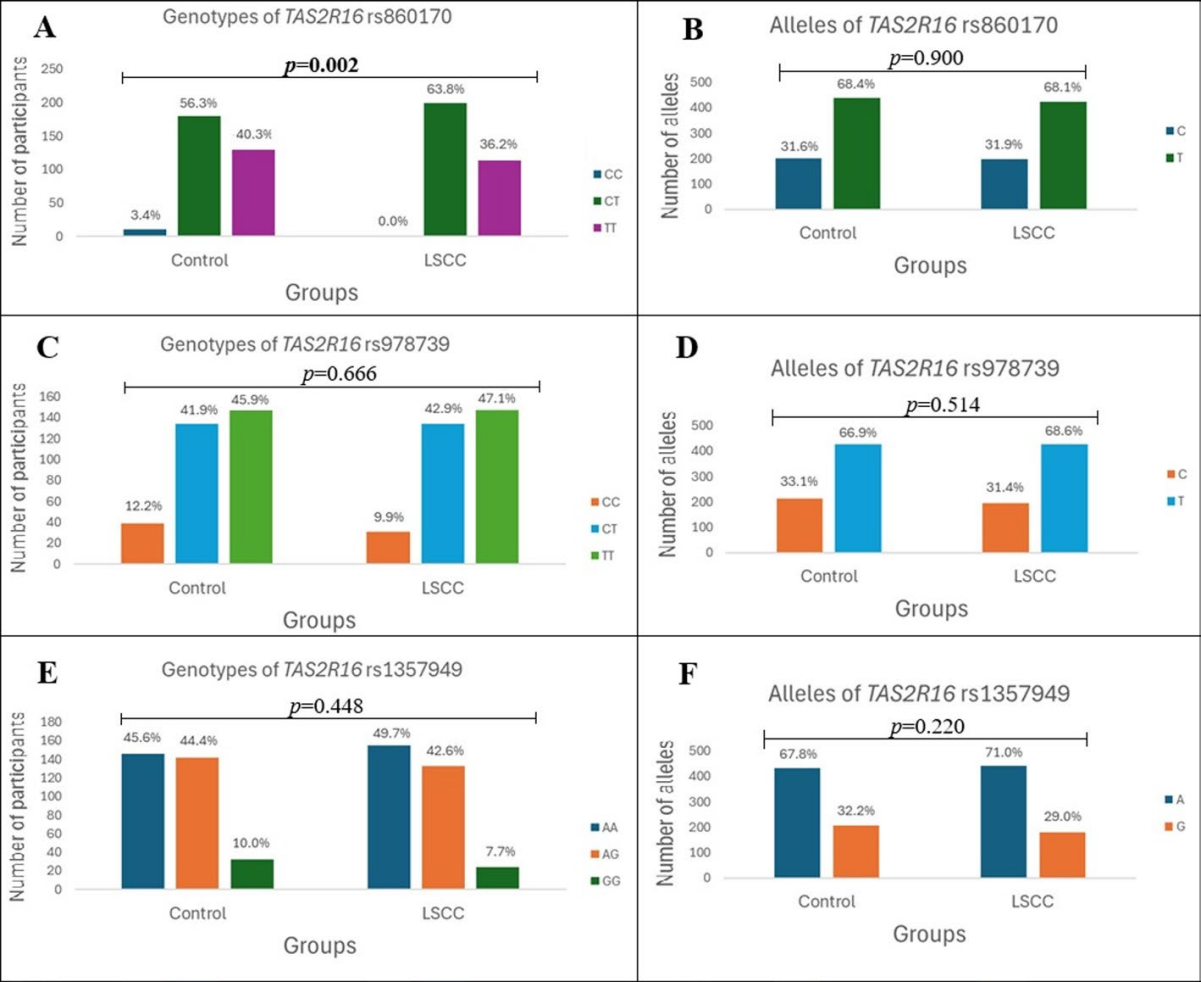
Genotype and allele frequencies of TAS2R16 in patients with LSCC and controls. A and B genotype and allele distributions of *TAS2R16* rs860170. C and D – genotype and allele distributions of *TAS2R16* rs978739. E and F – genotype and allele distributions of *TAS2R16* rs1357949. LSCC – laryngeal squamous cell carcinoma; *p*-value – significance level and Bonferroni corrected significance level when *p* = 0.05/3; the bolded results indicate significant differences between the groups

After analyzing the distribution of SNP genotypes and alleles, we found that the distribution of TT, CT, and CC genotypes of the *TAS2R16* rs860170 was statistically significantly different in LSCC patients compared to the control group (36.2%, 63.8%, and 0% vs. 40.3%, 56.3%, and 3.4%, *p* = 0.002) (Fig. 1).

Also, a binomial logistic regression analysis was performed to evaluate the influence of *TAS2R16* SNPs on the development of LSCC, but statistically significant associations were not found (Table 2).

**Table 2:** Binomial logistic regression analysis of *TAS2R16* (rs860170, rs978739, rs1357949) in patients with LSCC and controls

Model	Genotype/allele	OR (95% CI)	*p*–value	AIC
*TAS2R16* rs860170				
Codominant	CT vs. TT CC vs. TT	1.262 (0.914–1.744) –	0.158 –	862.877
Dominant	CC + CT vs. TT	1.189 (0.863–1.640)	0.290	876.915
Overdominant	CT vs. TT + CC	1.370 (0.995–1.885)	0.054	874.298
Recessive	CC vs. CT + TT	–	–	–
Additive	C	1.025 (0.758–1.386)	0.873	878.011
*TAS2R16* rs978739				
Codominant	CT vs. TT CC vs. TT	1.000 (0.718–1.392) 0.795 (0.471–1.342)	1.000 0.391	879.222
Dominant	CC + CT vs. TT	0.954 (0.698–1.304)	0.767	877.949
Overdominant	CT vs. TT + CC	1.045 (0.762–1.433)	0.785	877.962
Recessive	CC vs. CT + TT	0.795 (0.482–1.310)	0.368	877.222
Additive	C	0.927 (0.734–1.169)	0.521	877.624
*TAS2R16* rs1357949				
Codominant	AG vs. AA GG vs. AA	0.882 (0.636–1.224) 0.706 (0.397–1.256)	0.453 0.237	878.428
Dominant	GG + AG vs. AA	0.850 (0.622–1.162)	0.308	876.995
Overdominant	AG vs. AA + GG	0.931 (0.680–1.276)	0.658	877.841
Recessive	GG vs. AG + AA	0.750 (0.431–1.305)	0.309	876.991
Additive	G	0.857 (0.672–1.093)	0.857	876.493

OR – odds ratio, AIC – Akaike information criteria; the underlined AIC value indicates the best genetic model; CI – confidence interval; *p*-value – significance level; Bonferroni corrected significance level when *p* = 0.05/3

### Associations of *TAS2R16* rs860170, rs978739, rs1357949 SNPs with the clinical features of LSCC patients

**Fig. 2:**
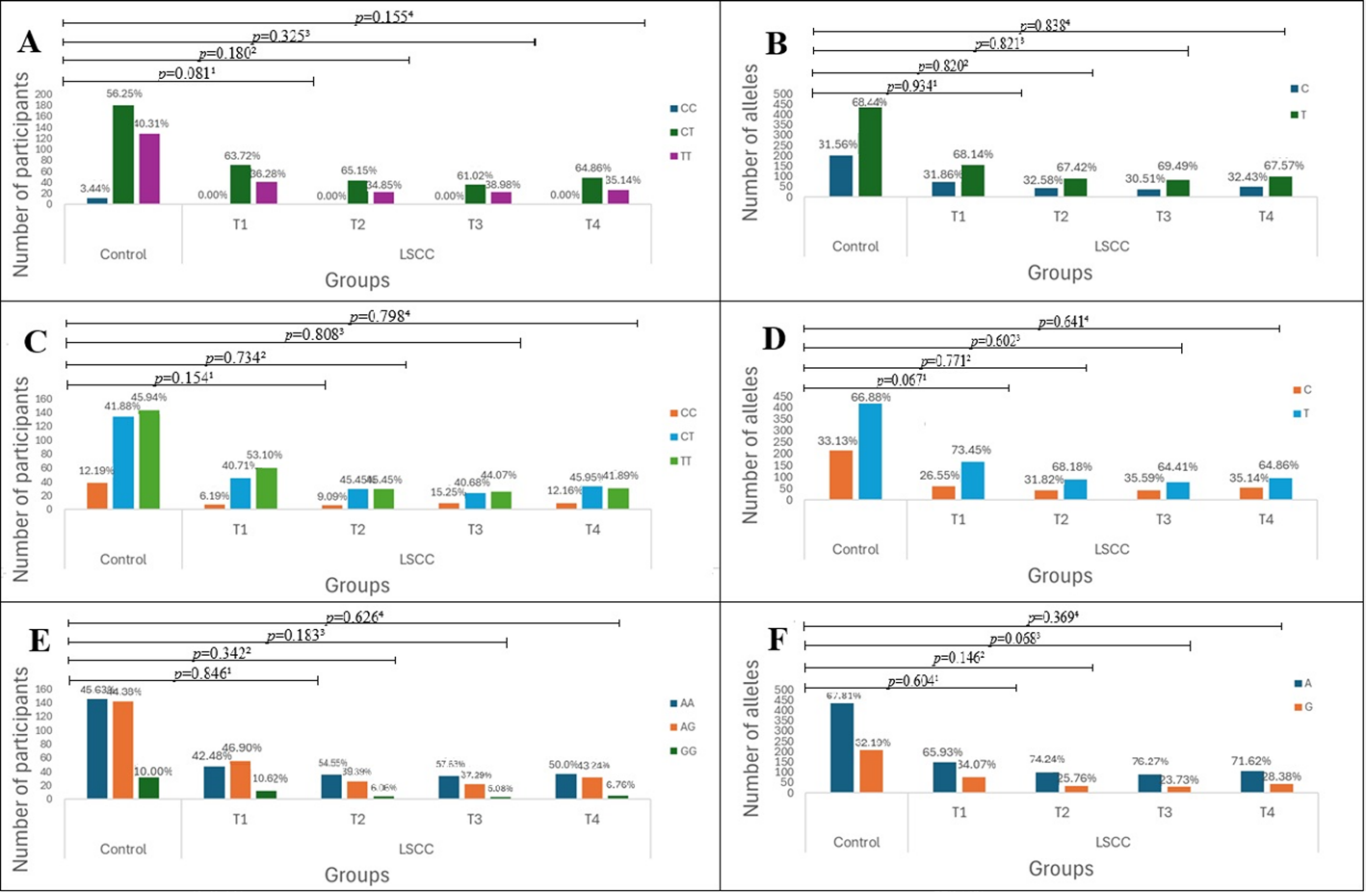
Genotype and allele frequencies of *TAS2R16* (rs860170, rs978739, rs1357949) in LSCC patients with different tumour sizes and in controls. A – genotype distribution of *TAS2R16* rs860170; B – allele distribution of *TAS2R16* rs860170; C – genotype distribution of *TAS2R16* rs978739; D – allele distribution of *TAS2R16* rs978739; E – genotype distribution of *TAS2R16* rs1357949; F – allele distribution of *TAS2R16* rs1357949. LSCC – laryngeal squamous cell carcinoma; *p*-value – significance level and Bonferroni corrected significance level when *p* = 0.05/3; *p*-value^1^ – control group vs. LSCC patients with T1 tumour size; *p*-value^2^ – control group vs. LSCC patients with T2 tumour size; *p*-value^3^ – control group vs. LSCC patients with T3 tumour size; *p*-value^4^ – control group vs. LSCC patients with T4 tumour size

Based on the clinical characteristics of LSCC, patients were categorized into four subgroups according to tumor size: T1, T2, T3, and T4. The frequencies of *TAS2R16* rs860170, rs978739, and rs1357949 genotypes and alleles were compared between patients with different LSCC sizes and control groups (Fig. 2).

Also, the binomial logistic regression analysis was performed with different tumour sizes. No statistically significant results were found (Tables 3, 4, 5 and 6).

**Table 3:** Binomi﻿al logistic regression analysis of *TAS2R16* (rs860170, rs978739, rs1357949) in LSCC patients with T1 tumour size and in controls

Model	Genotype/allele	OR (95% CI)	*p*–value	AIC
*TAS2R16* rs860170				
Codominant	CT vs. TT CC vs. TT	1.259 (0.806–1.964) –	0.311 –	493.355
Dominant	CC + CT vs. TT	1.186 (0.761–1.849)	0.451	498.572
Overdominant	CT vs. TT + CC	1.366 (0.877–2.126)	0.167	497.211
Recessive	CC vs. CT + TT	–	–	–
Additive	C	1.021 (0.682–1.528)	0.919	499.133
*TAS2R16* rs978739				
Codominant	CT vs. TT CC vs. TT	0.841 (0.536–1.319) 0.440 (0.186–1.038)	0.451 0.061	497.080
Dominant	CC + CT vs. TT	0.751 (0.488–1.154)	0.191	497.429
Overdominant	CT vs. TT + CC	0.953 (0.616–1.474)	0.829	499.097
Recessive	CC vs. CT + TT	0.476 (0.206–1.097)	0.081	495.651
Additive	C	0.737 (0.527–1.029)	0.073	495.830
*TAS2R16* rs1357949				
Codominant	AG vs. AA GG vs. AA	1.135 (0.721–1.787) 1.141 (0.545–2.389)	0.584 0.727	450.808
Dominant	GG + AG vs. AA	1.136 (0.737–1.752)	0.563	498.809
Overdominant	AG vs. AA + GG	1.107 (0.720–1.703)	0.643	498.929
Recessive	GG vs. AG + AA	1.069 (0.530–2.156)	0.851	499.109
Additive	G	1.091 (0.788–1.510)	0.600	498.869

OR – odds ratio, AIC – Akaike information criteria; the underlined AIC value indicates the best genetic model; CI – confidence interval; *p*-value – significance level; Bonferroni corrected significance level when *p* = 0.05/3

**Table 4:** Binomial logistic regression analysis of *TAS2R16* (rs860170, rs978739, rs1357949) in LSCC patients with T2 tumour size and in controls

Model	Genotype/allele	OR (95% CI)	*p*–value	AIC
*TAS2R16* rs860170				
Codominant	CT vs. TT CC vs. TT	1.340 (0.770–2.333) –	0.301 –	351.866
Dominant	CC + CT vs. TT	1.263 (0.726–2.196)	0.409	354.454
Overdominant	CT vs. TT + CC	1.454 (0.837–2.526)	0.184	353.341
Recessive	CC vs. CT + TT	–	–	–
Additive	C	1.073 (0.655–1.757)	0.780	355.069
*TAS2R16* rs978739				
Codominant	CT vs. TT CC vs. TT	1.097 (0.628–1.916) 0.754 (0.293–1.939)	0.745 0.558	356.502
Dominant	CC + CT vs. TT	1.020 (0.599–1.736)	0.943	355.141
Overdominant	CT vs. TT + CC	1.157 (0.679–1.971)	0.592	354.861
Recessive	CC vs. CT + TT	0.721 (0.292–1.779)	0.477	354.608
Additive	C	0.944 (0.636–1.401)	0.775	355.065
*TAS2R16* rs1357949				
Codominant	AG vs. AA GG vs. AA	0.743 (0.426–1.293) 0.507 (0.168–1.525)	0.293 0.227	354.924
Dominant	GG + AG vs. AA	0.699 (0.411–1.190)	0.188	353.402
Overdominant	AG vs. AA + GG	0.815 (0.474–1.399)	0.458	354.591
Recessive	GG vs. AG + AA	0.581 (0.198–1.701)	0.322	354.039
Additive	G	0.727 (0.473–1.116)	0.145	352.938

OR – odds ratio, AIC – Akaike information criteria; the underlined AIC value indicates the best genetic model; CI – confidence interval; *p*-value – significance level; Bonferroni corrected significance level when *p* = 0.05/3

**Table 5:** Binomial logistic regression analysis of *TAS2R16* (rs860170, rs978739, rs1357949) in LSCC patients with T3 tumour size and in controls

Model	Genotype/allele	OR (95% CI)	*p*–value	AIC
*TAS2R16* rs860170				
Codominant	CT vs. TT CC vs. TT	1.122 (0.634–1.983) –	0.693 –	327.838
Dominant	CC + CT vs. TT	1.057 (0.599–1.867)	0.848	329.741
Overdominant	CT vs. TT + CC	1.217 (0.690–2.148)	0.497	329.312
Recessive	CC vs. CT + TT	–	–	–
Additive	C	0.930 (0.556–1.556)	0.783	329.702
*TAS2R16* rs978739				
Codominant	CT vs. TT CC vs. TT	1.013 (0.555–1.849) 1.305 (0.565–3.011)	0.967 0.533	331.371
Dominant	CC + CT vs. TT	1.078 (0.617–1.886)	0.791	329.707
Overdominant	CT vs. TT + CC	0.952 (0.541–1.675)	0.864	329.748
Recessive	CC vs. CT + TT	1.297 (0.592–2.843)	0.516	329.372
Additive	C	1.108 (0.744–1.651)	0.613	329.524
*TAS2R16* rs1357949				
Codominant	AG vs. AA GG vs. AA	0.665 (0.371–1.193) 0.403 (0.116–1.392)	0.171 0.151	328.230
Dominant	GG + AG vs. AA	0.617 (0.352–1.081)	0.092	326.899
Overdominant	AG vs. AA + GG	0.745 (0.421–1.320)	0.314	328.747
Recessive	GG vs. AG + AA	0.482 (0.143–1.629)	0.240	328.132
Additive	G	0.651 (0.410–1.032)	0.068	326.245

OR – odds ratio, AIC – Akaike information criteria; the underlined AIC value indicates the best genetic model; CI – confidence interval; *p*-value – significance level; Bonferroni corrected significance level when *p* = 0.05/3

**Table 6:** Binomial logistic regression analysis of *TAS2R16* (rs860170, rs978739, rs1357949) in LSCC patients with T4 tumour size and in controls

Model	Genotype/allele	OR (95% CI)	*p*–value	AIC
*TAS2R16* rs860170				
Codominant	CT vs. TT CC vs. TT	1.323 (0.780–2.244) –	0.299 –	378.891
Dominant	CC + CT vs. TT	1.247 (0.736–2.112)	0.412	381.955
Overdominant	CT vs. TT + CC	1.436 (0.849–2.429)	0.178	380.781
Recessive	CC vs. CT + TT	–	–	–
Additive	C	1.062 (0.663–1.702)	0.801	382.574
*TAS2R16* rs978739				
Codominant	CT vs. TT CC vs. TT	1.203 (0.701–2.065) 1.094 (0.481–2.489)	0.502 0.830	384.186
Dominant	CC + CT vs. TT	1.179 (0.707–1.966)	0.529	382.239
Overdominant	CT vs. TT + CC	1.180 (0.710–1.961)	0.524	382.232
Recessive	CC vs. CT + TT	0.998 (0.460–2.162)	0.995	382.637
Additive	C	1.090 (0.754–1.574)	0.648	382.430
*TAS2R16* rs1357949				
Codominant	AG vs. AA GG vs. AA	0.889 (0.525–1.506) 0.617 (0.225–1.691)	0.662 0.348	383.647
Dominant	GG + AG vs. AA	0.839 (0.506–1.392)	0.497	382.176
Overdominant	AG vs. AA + GG	0.955 (0.573–1.591)	0.860	382.606
Recessive	GG vs. AG + AA	0.652 (0.245–1.735)	0.392	381.838
Additive	G	0.831 (0.558–1.239)	0.363	381.797

OR – odds ratio, AIC – Akaike information criteria; the underlined AIC value indicates the best genetic model; CI – confidence interval; *p*-value – significance level; Bonferroni corrected significance level when *p* = 0.05/3

**Fig. 3:**
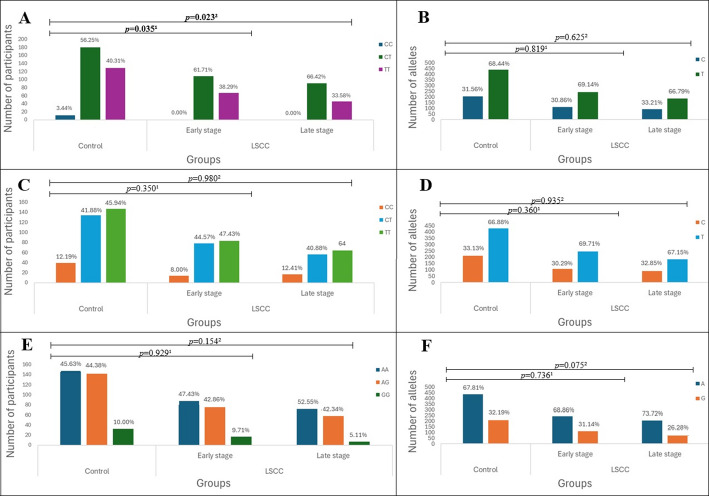
Genotype and allele frequencies of *TAS2R16* (rs860170, rs978739, rs1357949) in LSCC patients and controls according to the stage of the LSCC. A – genotype distribution of *TAS2R16* rs860170; B – allele distribution of *TAS2R16* rs860170; C – genotype distribution of *TAS2R16* rs978739; D – allele distribution of *TAS2R16* rs978739; E – genotype distribution of *TAS2R16* rs1357949; F – allele distribution of *TAS2R16* rs1357949; LSCC – laryngeal squamous cell carcinoma; *p*-value – significance level and Bonferroni corrected significance level when *p* = 0.05/3; *p*-value^1^ – control group vs. early-stage LSCC patients; *p*-value^2^ – control group vs. late-stage LSCC patients; the bolded results indicate significant differences between the groups

Based on the stages of the LSCC, patients were categorized into early and late stages. The results showed that the distribution of TT, CT, and CC genotypes of *TAS2R16* rs860170 is statistically significantly different in both early-stage and late-stage LSCC patients compared to the control group (38.29%, 61.71%, and 0.00% vs. 40.31%, 56.25%, and 3.44%, *p* = 0.035, 33.58%, 66.42%, and 0.00% vs. 40.31%, 56.25%, and 3.44%, *p* = 0.023, respectively). When we applied Bonferroni’s corrected significance threshold, these results did not reach statistical significance. (Fig. 3).

Binomial logistic regression analysis does not reveal statistically significant results in the early-stage group (Table 7).

**Table 7:** Binomial logistic regression analysis of *TAS2R16* (rs860170, rs978739, rs1357949) in early-stage LSCC patients and controls

Model	Genotype/allele	OR (95% CI)	*p*–value	AIC
*TAS2R16* rs860170				
Codominant	CT vs. TT CC vs. TT	1.155 (0.790–1.688) –	0.456 –	636.821
Dominant	CC + CT vs. TT	1.089 (0.746–1.589)	0.659	644.917
Overdominant	CT vs. TT + CC	1.254 (0.860–1.827)	0.239	643.717
Recessive	CC vs. CT + TT	–	–	–
Additive	C	0.951 (0.670–1.348)	0.776	645.031
*TAS2R16* rs978739				
Codominant	CT vs. TT CC vs. TT	1.031 (0.700–1.519) 0.636 (0.326–1.239)	0.878 0.183	644.926
Dominant	CC + CT vs. TT	0.942 (0.651–1.363)	0.751	645.011
Overdominant	CT vs. TT + CC	1.116 (0.770–1.619)	0.562	644.776
Recessive	CC vs. CT + TT	0.627 (0.330–1.189)	0.153	642.950
Additive	C	0.879 (0.665–1.162)	0.365	644.285
*TAS2R16* rs1357949				
Codominant	AG vs. AA GG vs. AA	0.929 (0.630–1.370) 0.934 (0.489–1.785)	0.710 0.837	646.936
Dominant	GG + AG vs. AA	0.930 (0.643–1.346)	0.700	644.964
Overdominant	AG vs. AA + GG	0.940 (0.648–1.364)	0.745	645.006
Recessive	GG vs. AG + AA	0.968 (0.521–1.799)	0.919	645.101
Additive	G	0.952 (0.718–1.263)	0.735	644.996

OR – odds ratio, AIC – Akaike information criteria; the underlined AIC value indicates the best genetic model; CI – confidence interval; *p*-value – significance level; Bonferroni corrected significance level when *p* = 0.05/3

Binomial logistic regression analysis revealed that *TAS2R16* rs860170 CT genotype is associated with 1.5-fold increase odds of developing late-stage LSCC under the overdominant model (OR = 1.539, 95% CI: 1.013–2.336, *p* = 0.043), although, this result does not survive Bonferroni correction (Table 8).

**Table 8:** Binomial logistic regression analysis of *TAS2R16* (rs860170, rs978739, rs1357949) in late-stage LSCC patients and controls

Model	Genotype/allele	OR (95% CI)	*p*–value	AIC
*TAS2R16* rs860170				
Codominant	CT vs. TT CC vs. TT	1.418 (0.931–2.159) –	0.104 –	551.516
Dominant	CC + CT vs. TT	1.336 (0.879–2.032)	0.175	558.299
Overdominant	CT vs. TT + CC	1.539 (1.013–2.336)	**0.043**	555.990
Recessive	CC vs. CT + TT	–	–	–
Additive	C	1.126 (0.770–1.647)	0.540	559.785
*TAS2R16* rs978739				
Codominant	CT vs. TT CC vs. TT	0.960 (0.625–1.473) 1.001 (0.528–1.900)	0.851 0.997	562.121
Dominant	CC + CT vs. TT	0.969 (0.649–1.448)	0.879	560.137
Overdominant	CT vs. TT + CC	0.960 (0.639–1.441)	0.843	560.121
Recessive	CC vs. CT + TT	1.021 (0.556–1.876)	0.947	560.156
Additive	C	0.988 (0.738–1.324)	0.937	560.154
*TAS2R16* rs1357949				
Codominant	AG vs. AA GG vs. AA	0.828 (0.546–1.255) 0.444 (0.187–1.054)	0.374 0.066	558.155
Dominant	GG + AG vs. AA	0.758 (0.507–1.131)	0.175	558.315
Overdominant	AG vs. AA + GG	0.920 (0.614–1.379)	0.687	559.998
Recessive	GG vs. AG + AA	0.485 (0.208–1.127)	0.092	556.946
Additive	G	0.743 (0.538–1.026)	0.071	556.825

OR – odds ratio, AIC – Akaike information criteria; the underlined AIC value indicates the best genetic model; CI – confidence interval; *p*-value – significance level; Bonferroni corrected significance level when *p* = 0.05/3; the bolded results indicate significant differences between the groups

**Fig. 4:**
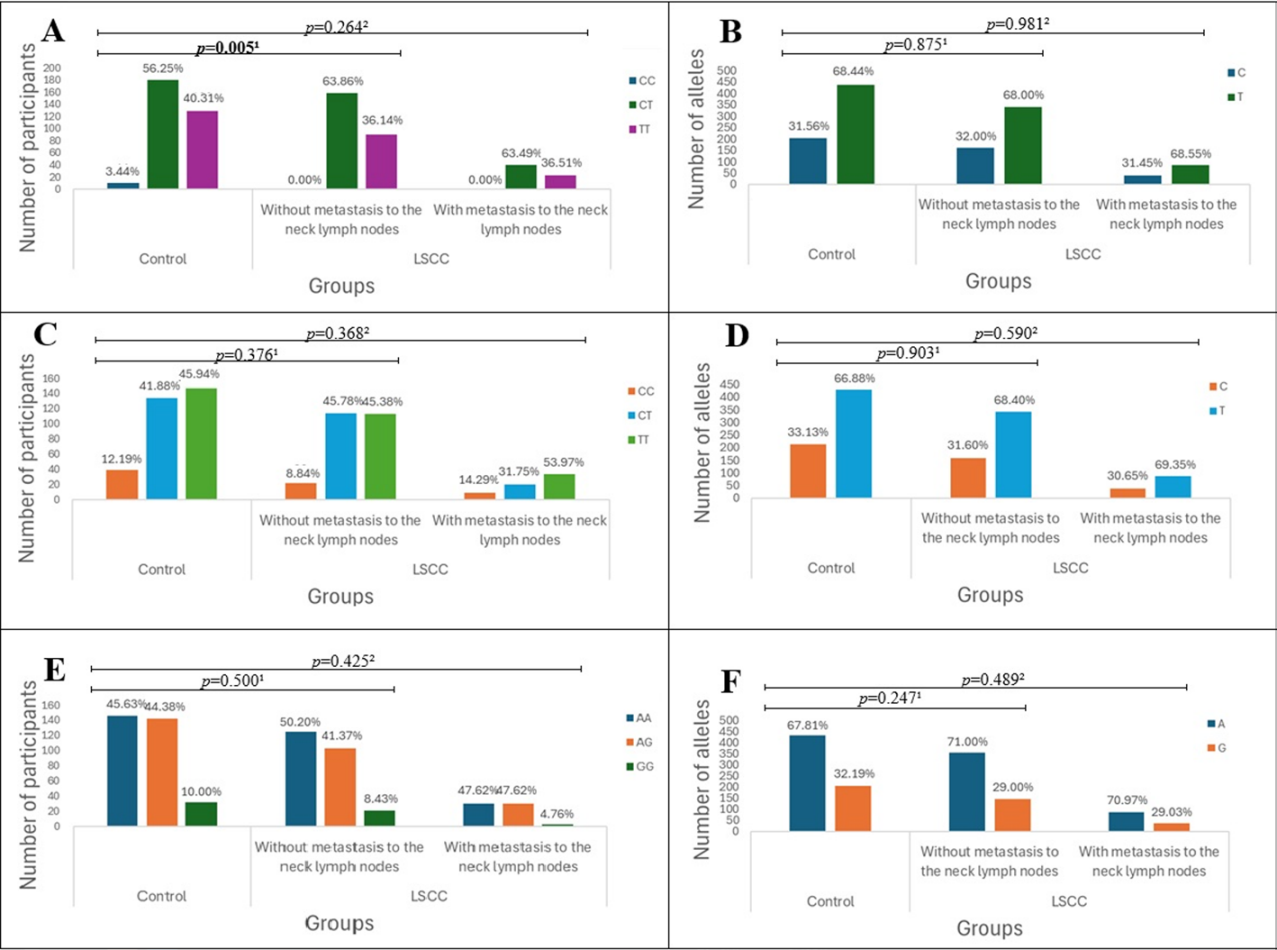
Genotype and allele frequencies of *TAS2R16* (rs860170, rs978739, rs1357949) in LSCC patients and controls according to metastasis to the neck lymph nodes. A – genotype distribution of *TAS2R16* rs860170; B – allele distribution of *TAS2R16* rs860170; C – genotype distribution of *TAS2R16* rs978739; D – allele distribution of *TAS2R16* rs978739; E – genotype distribution of *TAS2R16* rs1357949; F – allele distribution of *TAS2R16* rs1357949; LSCC – laryngeal squamous cell carcinoma; *p*-value – significance level and Bonferroni corrected significance level when *p* = 0.05/3; *p*-value^1^ – control group vs. LSCC patients without metastasis; *p*-value^2^ – control group vs. LSCC patients with metastasis; the bolded results indicate significant differences between the groups

The impact of the selected SNPs on LSCC progression was analyzed by categorizing LSCC patients based on the presence of regional lymph node metastasis: patients without metastasis (N0) and those with metastasis to neck lymph nodes (N1-N3). A statistically significant difference was observed in the distribution of TT, CT, and CC genotypes of *TAS2R16* rs860170 between LSCC patients without metastasis and the control group (36.14%, 63.86%, and 0.00% vs. 40.31%, 56.25%, and 3.44%, *p* = 0.005) (Fig. 4).

Binomial logistic regression analysis of rs860170, rs978739, and rs1357949 SNPs in LSCC patients without and with neck lymph node metastases and controls did not reveal any statistically significant results (Tables 9 and 10).

**Table 9:** Binomial logistic regression analysis of *TAS2R16* (rs860170, rs978739, rs1357949) in LSCC patients without metastasis to the neck lymph nodes and in controls

Model	Genotype/allele	OR (95% CI)	*p*–value	AIC
*TAS2R16* rs860170				
Codominant	CT vs. TT CC vs. TT	1.274 (0.904–1.796) –	0.167 –	770.779
Dominant	CC + CT vs. TT	1.201 (0.853–1.689)	0.294	782.464
Overdominant	CT vs. TT + CC	1.383 (0.984–1.942)	0.062	780.054
Recessive	CC vs. CT + TT	–	–	–
Additive	C	1.033 (0.751–1.420)	0.842	783.530
*TAS2R16* rs978739				
Codominant	CT vs. TT CC vs. TT	1.097 (0.773–1.556) 0.727 (0.408–1.295)	0.604 0.280	783.589
Dominant	CC + CT vs. TT	1.014 (0.727–1.413)	0.936	783.563
Overdominant	CT vs. TT + CC	1.164 (0.834–1.624)	0.374	782.778
Recessive	CC vs. CT + TT	0.695 (0.401–1.206)	0.196	781.858
Additive	C	0.933 (0.727–1.198)	0.587	783.274
*TAS2R16* rs1357949				
Codominant	AG vs. AA GG vs. AA	0.840 (0.593–1.190) 0.760 (0.417–1.385)	0.328 0.371	784.182
Dominant	GG + AG vs. AA	0.826 (0.593–1.150)	0.258	782.287
Overdominant	AG vs. AA + GG	0.878 (0.628–1.228)	0.447	782.992
Recessive	GG vs. AG + AA	0.825 (0.463–1.470)	0.514	783.140
Additive	G	0.859 (0.665–1.110)	0.246	782.215

OR – odds ratio, AIC – Akaike information criteria; the underlined AIC value indicates the best genetic model; CI – confidence interval; *p*-value – significance level; Bonferroni corrected significance level when *p* = 0.05/3

**Table 10:** Binomial logistic regression analysis of *TAS2R16* (rs860170, rs978739, rs1357949) in LSCC patients with metastasis to the neck lymph nodes and in controls

Model	Genotype/allele	OR (95% CI)	*p*–value	AIC
*TAS2R16* rs860170				
Codominant	CT vs. TT CC vs. TT	1.215 (0.692–2.133) –	0.497 –	338.386
Dominant	CC + CT vs. TT	1.145 (0.653–2.008)	0.636	340.586
Overdominant	CT vs. TT + CC	1.319 (0.753–2.310)	0.333	339.861
Recessive	CC vs. CT + TT	–	–	–
Additive	C	0.992 (0.599–1.644)	0.976	340.810
*TAS2R16* rs978739				
Codominant	CT vs. TT CC vs. TT	0.665 (0.364–1.215) 1.028 (0.454–2.328)	0.184 0.947	340.766
Dominant	CC + CT vs. TT	0.747 (0.433–1.288)	0.294	339.706
Overdominant	CT vs. TT + CC	0.661 (0.371–1.177)	0.160	338.771
Recessive	CC vs. CT + TT	1.224 (0.560–2.674)	0.613	340.563
Additive	C	0.900 (0.603–1.342)	0.605	340.541
*TAS2R16* rs1357949				
Codominant	AG vs. AA GG vs. AA	1.064 (0.607–1.863) 0.472 (0.135–1.645)	0.829 0.239	340.846
Dominant	GG + AG vs. AA	0.955 (0.554–1.647)	0.868	340.784
Overdominant	AG vs. AA + GG	1.175 (0.682–2.026)	0.561	340.474
Recessive	GG vs. AG + AA	0.458 (0.136–1.544)	0.208	338.892
Additive	G	0.857 (0.557–1.317)	0.481	340.307

OR – odds ratio, AIC – Akaike information criteria; the underlined AIC value indicates the best genetic model; CI – confidence interval; *p*-value – significance level; Bonferroni corrected significance level when *p* = 0.05/3

**Fig. 5:**
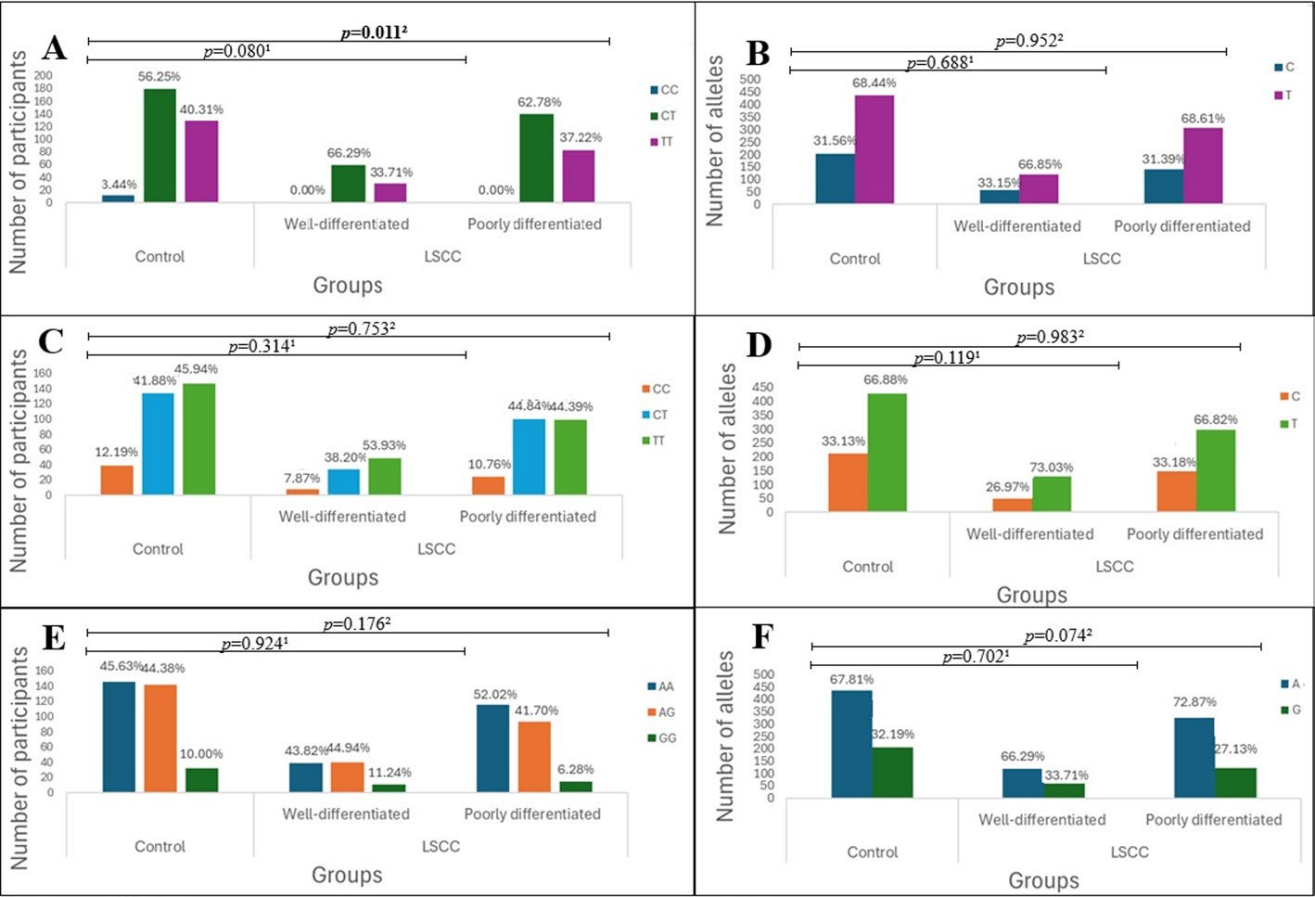
Genotype and allele frequencies of *TAS2R16* (rs860170, rs978739, rs1357949) in LSCC patients and controls according to the differentiation of cancer cells. A – genotype distribution of *TAS2R16* rs860170; B – allele distribution of *TAS2R16* rs860170; C – genotype distribution of *TAS2R16* rs978739; D – allele distribution of *TAS2R16* rs978739; E – genotype distribution of *TAS2R16* rs1357949; F – allele distribution of *TAS2R16* rs1357949; LSCC – laryngeal squamous cell carcinoma; *p*-value – significance level and Bonferroni corrected significance level when *p* = 0.05/3; *p*-value^1^ – control group vs. LSCC patients with well-differentiated cancer cells; *p*-value^2^ – control group vs. LSCC patients with poorly differentiated cancer cells; the bolded results indicate significant differences between the groups

The genotypes and alleles of *TAS2R16* rs860170, rs978739, and rs1357949 were examined concerning the differentiation level of LSCC cells. Based on the patient’s clinical data, LSCC cell differentiation was categorized as well-differentiated (G1) and poorly differentiated (G2–G3). A statistically significant difference was identified in the distribution of TT, CT, and CC genotypes of *TAS2R16* rs860170 between patients with poorly differentiated LSCC and the control group (37.22%, 62.78%, and 0.00% vs. 40.31%, 56.25%, 3.44%, *p* = 0.011) (Fig. 5).

Binomial logistic regression analysis does not reveal any statistically significant results (Tables 11 and 12).

**Table 11:** Binomial logistic regression analysis of *TAS2R16* (rs860170, rs978739, rs1357949) in LSCC patients with well-differentiated cells and in controls

Model	Genotype/allele	OR (95% CI)	*p*–value	AIC
*TAS2R16* rs860170				
Codominant	CT vs. TT CC vs. TT	1.409 (0.860–2.310) –	0.174 –	423.144
Dominant	CC + CT vs. TT	1.328 (0.811–2.175)	0.259	429.221
Overdominant	CT vs. TT + CC	1.530 (0.935–2.502)	0.090	427.575
Recessive	CC vs. CT + TT	–	–	–
Additive	C	1.118 (0.719–1.737)	0.620	430.271
*TAS2R16* rs978739				
Codominant	CT vs. TT CC vs. TT	0.777 (0.472–1.278) 0.550 (0.231–1.309)	0.321 0.177	430.120
Dominant	CC + CT vs. TT	0.726 (0.453–1.163)	0.183	428.733
Overdominant	CT vs. TT + CC	0.858 (0.530–1.389)	0.534	430.126
Recessive	CC vs. CT + TT	0.615 (0.265–1.427)	0.258	429.114
Additive	C	0.756 (0.526–1.085)	0.129	428.146
*TAS2R16* rs1357949				
Codominant	AG vs. AA GG vs. AA	1.055 (0.641–1.735) 1.170 (0.529–2.586)	0.834 0.698	432.359
Dominant	GG + AG vs. AA	1.076 (0.670–1.726)	0.762	430.425
Overdominant	AG vs. AA + GG	1.023 (0.638–1.641)	0.924	430.507
Recessive	GG vs. AG + AA	1.139 (0.537–2.418)	0.734	430.403
Additive	G	1.072 (0.752–1.529)	0.700	430.368

OR – odds ratio, AIC – Akaike information criteria; the underlined AIC value indicates the best genetic model; CI – confidence interval; *p*-value – significance level; Bonferroni corrected significance level when *p* = 0.05/3

**Table 12:** Binomial logistic regression analysis of *TAS2R16* (rs860170, rs978739, rs1357949) in LSCC patients with poorly differentiated cells and controls

Model	Genotype/allele	OR (95% CI)	*p*–value	AIC
*TAS2R16* rs860170				
Codominant	CT vs. TT CC vs. TT	1.209 (0.849–1.721) –	0.293 –	726.435
Dominant	CC + CT vs. TT	1.139 (0.802–1.619)	0.467	736.807
Overdominant	CT vs. TT + CC	1.312 (0.924–1.862)	0.128	735.012
Recessive	CC vs. CT + TT	–	–	–
Additive	C	0.987 (0.712–1.369)	0.940	737.331
*TAS2R16* rs978739				
Codominant	CT vs. TT CC vs. TT	1.108 (0.771–1.594) 0.914 (0.517–1.614)	0.580 0.756	738.768
Dominant	CC + CT vs. TT	1.064 (0.755–1.501)	0.722	737.210
Overdominant	CT vs. TT + CC	1.129 (0.799–1.593)	0.492	736.865
Recessive	CC vs. CT + TT	0.869 (0.506–1.491)	0.610	737.075
Additive	C	1.003 (0.778–1.292)	0.984	737.336
*TAS2R16* rs1357949				
Codominant	AG vs. AA GG vs. AA	0.824 (0.577–1.179) 0.551 (0.281–1.080)	0.289 0.083	735.788
Dominant	GG + AG vs. AA	0.774 (0.549–1.090)	0.143	735.185
Overdominant	AG vs. AA + GG	0.897 (0.635–1.267)	0.537	736.954
Recessive	GG vs. AG + AA	0.603 (0.314–1.158)	0.129	734.912
Additive	G	0.778 (0.593–1.021)	0.070	734.023

OR – odds ratio, AIC – Akaike information criteria; the underlined AIC value indicates the best genetic model; CI – confidence interval; *p*-value – significance level; Bonferroni corrected significance level when *p* = 0.05/3

### Associations of *TAS2R16* rs860170, rs978739, rs1357949 SNPs with the lifestyle habits of LSCC patients

**Fig. 6:**
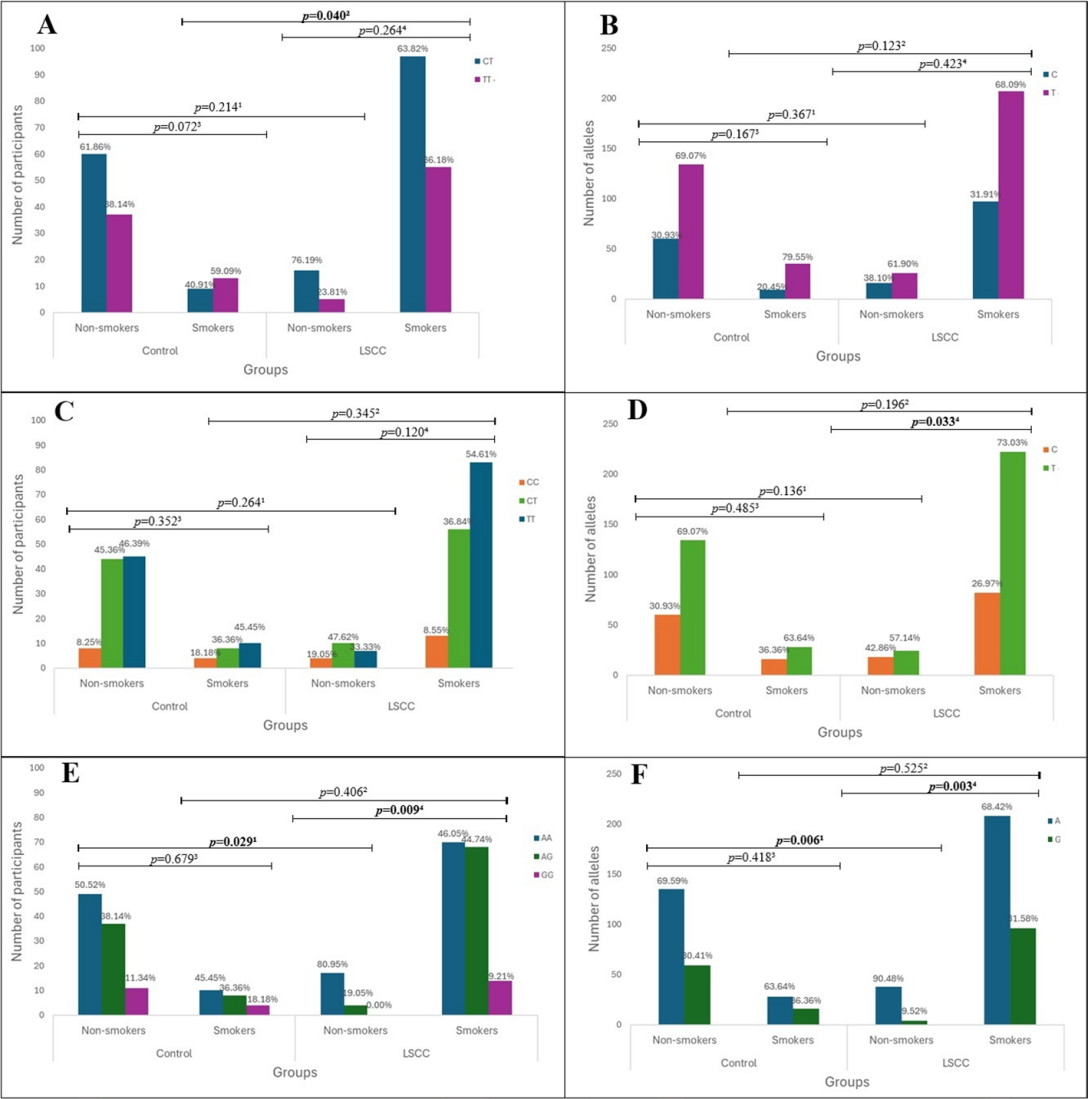
Genotype and allele frequencies of *TAS2R16* (rs860170, rs978739, rs1357949) in LSCC patients and controls according to their smoking habits. A – genotype distribution of *TAS2R16* rs860170; B – allele distribution of *TAS2R16* rs860170; C – genotype distribution of *TAS2R16* rs978739; D – allele distribution of *TAS2R16* rs978739; E – genotype distribution of *TAS2R16* rs1357949; F – allele distribution of *TAS2R16* rs1357949; LSCC – laryngeal squamous cell carcinoma; *p*-value – significance level and Bonferroni corrected significance level when *p* = 0.05/3; the bolded results indicate significant differences between the groups; *p*-value^1^ – control group non-smokers vs. LSCC group non-smokers; *p*-value^2^ – control group smokers vs. LSCC group smokers; *p*-value^3^ - control group non-smokers vs. smokers; *p*-value^4^ – LSCC group non-smokers vs. smokers; the bolded results indicate significant differences between the groups

According to the smoking habits of the study subjects, all participants were divided into groups: smokers and non-smokers. Results showed that the distribution of the *TAS2R16* rs860170 TT, CT, and CC genotypes was statistically significantly different between smoking controls and LSCC patients who smoked (59.09%, 40.91%, and 0.00% vs. 36.16%, 63.82%, and 0.00%, *p* = 0.040). *TAS2R16* rs978739 C allele is more frequent in non-smokers than in smokers of the LSCC group (42.86% vs. 26.97%, *p* = 0.033), although these results do not survive Bonferroni correction. However, the analysis of genotype and allele distribution revealed that *TAS2R16* rs1357949 AA, AG, and GG genotypes were statistically significantly different between non-smoking controls and non-smoking LSCC patients (50.52%, 38.14%, and 11.34% vs. 80.95%, 19.05%, and 0.00%, *p* = 0.029). Still, these results do not survive Bonferroni correction. Also, the distribution of these genotypes is statistically significantly different between non-smokers of the LSCC group and LSCC smokers (80.95%, 19.05%, and 0.00% vs. 46.05%, 44.74%, and 9.21%, *p* = 0.009). Results showed that the G allele of the *TAS2R16* rs1357949 is less common in non-smokers of the LSCC group than the non-smokers of the control group (9.52% vs. 30.41%, *p* = 0.006). Results showed that the rs1357949 G allele is less frequent in the non-smokers than in the smokers LSCC group (9.52% vs. 31.58%, *p* = 0.003). When comparing the control group smokers with the LSCC group smokers, the distribution of the genotypes and alleles showed no statistically significant results. (Fig. 6).

Binomial logistic regression analysis showed that *TAS2R16* rs1357949 GG and AG genotypes together are associated with 4.2-fold decreased odds of developing LSCC in non-smoking patients under the dominant model (OR = 0.240, 95% CI: 0.075–0.766, *p* = 0.016). Also, each rs1357949 G allele was found to decrease the odds of LSCC occurrence in non-smokers by 3.8-fold under the additive model (OR = 0.262, 95% CI: 0.090–0.763, *p* = 0.014) (Table 13).

**Table 13:** Binomial logistic regression analysis of *TAS2R16* (rs860170, rs978739, rs1357949) in non-smokers with LSCC and non-smoking controls

Model	Genotype/allele	OR (95% CI)	*p*–value	AIC
*TAS2R16* rs860170				
Codominant	CT vs. TT CC vs. TT	1.973 (0.667–5.837) –	0.219 –	112.889
Dominant	CC + CT vs. TT	1.973 (0.667–5.837)	0.219	110.889
Overdominant	CT vs. TT + CC	1.973 (0.667–5.837)	0.219	110.889
Recessive	CC vs. CT + TT	–	–	–
Additive	C	1.973 (0.667–5.837)	0.219	110.889
*TAS2R16* rs978739				
Codominant	CT vs. TT CC vs. TT	1.461 (0.511–4.181) 3.214 (0.761–13.572)	0.480 0.112	112.113
Dominant	CC + CT vs. TT	1.731 (0.642–4.663)	0.278	111.298
Overdominant	CT vs. TT + CC	1.095 (0.426–2.818)	0.851	112.482
Recessive	CC vs. CT + TT	2.618 (0.708–9.678)	0.149	110.619
Additive	C	1.718 (0.846–3.486)	0.134	110.276
*TAS2R16* rs1357949				
Codominant	AG vs. AA GG vs. AA	0.312 (0.097–1.004) –	0.051	105.521
Dominant	GG + AG vs. AA	0.240 (0.075–0.766)	**0.016**	105.510
Overdominant	AG vs. AA + GG	0.382 (0.119–1.222)	0.105	109.510
Recessive	GG vs. AG + AA	–	–	–
Additive	G	0.262 (0.090–0.763)	**0.014**	104.136

OR – odds ratio, AIC – Akaike information criteria; the underlined AIC value indicates the best genetic model; CI – confidence interval; *p*-value – significance level; Bonferroni corrected significance level when *p* = 0.05/3. The bolded results indicate significant differences between the groups

While binomial logistic regression analysis in smokers with LSCC patients and smoking controls showed that the *TAS2R16* rs860170 CT genotype was found to increase the odds of developing LSCC in smokers by 2.5 times under the codominant and overdominant models, while the CT + CC models increase these odds by 2.5-fold under the dominant model, also each C allele increases these odds by 2.5 times (OR = 2.547, 95% CI: 1.023–6.342, *p* = 0.044 in all models). Although these results do not survive Bonferroni correction (Table 14).

**Table 14:** Binomial logistic regression analysis of *TAS2R16* (rs860170, rs978739, rs1357949) in smokers with LSCC and smoking controls

Model	Genotype/allele	OR (95% CI)	*p*–value	AIC
*TAS2R16* rs860170				
Codominant	CT vs. TT CC vs. TT	2.547 (1.023–6.342) –	**0.044** –	131.963
Dominant	CC + CT vs. TT	2.547 (1.023–6.342)	**0.044**	129.963
Overdominant	CT vs. TT + CC	2.547 (1.023–6.342)	**0.044**	129.963
Recessive	CC vs. CT + TT	–	–	–
Additive	C	2.547 (1.023–6.342)	**0.044**	129.963
*TAS2R16* rs978739				
Codominant	CT vs. TT CC vs. TT	0.843 (0.314–2.269) 0.392 (0.107–1.435)	0.736 0.157	134.261
Dominant	CC + CT vs. TT	0.693 (0.282–1.700)	0.423	133.441
Overdominant	CT vs. TT + CC	1.021 (0.403–2.585)	0.965	134.084
Recessive	CC vs. CT + TT	0.421 (0.124–1.431)	0.166	132.374
Additive	C	0.670 (0.354–1.268)	0.219	132.617
*TAS2R16* rs1357949				
Codominant	AG vs. AA GG vs. AA	1.214 (0.452–3.261) 0.500 (0.137–1.823)	0.700 0.294	134.500
Dominant	GG + AG vs. AA	0.976 (0.398–2.396)	0.958	134.083
Overdominant	AG vs. AA + GG	1.417 (0.561–3.575)	0.461	133.530
Recessive	GG vs. AG + AA	0.457 (0.135–1.538)	0.206	132.649
Additive	G	0.807 (0.417–1.565)	0.526	133.689

OR – odds ratio, AIC – Akaike information criteria; the underlined AIC value indicates the best genetic model; CI – confidence interval; *p*-value – significance level; Bonferroni corrected significance level when *p* = 0.05/3. The bolded results indicate significant differences between the groups

The analysis of non-smokers and smoking controls did not show any statistically significant results either (Table 15).

**Table 15:** Binomial logistic regression analysis of *TAS2R16* (rs860170, rs978739, rs1357949) in non-smokers and smoking controls

Model	Genotype/allele	OR (95% CI)	*p*–value	AIC
*TAS2R16* rs860170				
Codominant	CT vs. TT CC vs. TT	0.427 (0.166–1.097) –	0.077	114.741
Dominant	CC + CT vs. TT	0.427 (0.166–1.097)	0.077	112.741
Overdominant	CT vs. TT + CC	0.427 (0.166–1.097)	0.077	112.741
Recessive	CC vs. CT + TT	–	–	–
Additive	C	0.427 (0.166–1.097)	0.077	112.741
*TAS2R16* rs978739				
Codominant	CT vs. TT CC vs. TT	0.818 (0.296–2.265) 2.250 (0.565–8.962)	0.699 0.250	116.081
Dominant	CC + CT vs. TT	1.038 (0.410–2.630)	0.937	115.925
Overdominant	CT vs. TT + CC	0.688 (0.265–1.791)	0.444	115.334
Recessive	CC vs. CT + TT	2.472 (0.672–9.096)	0.173	114.231
Additive	C	1.279 (0.641–2.548)	0.485	115.449
*TAS2R16* rs1357949				
Codominant	AG vs. AA GG vs. AA	1.059 (0.381–2.947) 1.782 (0.471–6.745)	0.912 0.395	117.217
Dominant	GG + AG vs. AA	1.225 (0.484–3.101)	0.668	115.748
Overdominant	AG vs. AA + GG	0.927 (0.355–2.421)	0.876	115.907
Recessive	GG vs. AG + AA	1.737 (0.497–6.077)	0.387	115.230
Additive	G	1.269 (0.664–2.425)	0.471	115.418

OR – odds ratio, AIC – Akaike information criteria; the underlined AIC value indicates the best genetic model; CI – confidence interval; *p*-value – significance level; Bonferroni corrected significance level when *p* = 0.05/3

Results revealed that *TAS2R16* rs978739 each C allele is associated with reduced odds of smoking for LSCC patients by 1.9-fold under the additive model (OR = 0.514, 95% CI: 0.269–0.983, *p* = 0.044), after applying Bonferroni’s corrected significance threshold, this result did not reach statistical significance. *TAS2R16* rs1357949 AG genotype was found to increase the odds of smoking for LSCC patients by 4.2-fold under the codominant model (OR = 4.129, 95% CI: 1.321–12.898, *p* = 0.015), while GG and AG genotypes together increase these odds by 5 times under the dominant model (OR = 4.979, 95% CI: 1.600-15.488, *p* = 0.006). According to the overdominant model, AG genotype is associated with increased odds of smoking for the LSCC group (OR = 3.440, 95% CI: 1.106–10.705, *p* = 0.033), but this result does not survive after Bonferroni correction. However, each G allele increases the odds of the smoking habit for LSCC patients by 4.6-fold under the additive model (OR = 4.570, 95% CI: 1.553–13.447, *p* = 0.006) (Table 16).

**Table 16:** Binomial logistic regression analysis of *TAS2R16* (rs860170, rs978739, rs1357949) in non-smokers and smoking LSCC patients

Model	Genotype/allele	OR (95% CI)	*p*–value	AIC
*TAS2R16* rs860170				
Codominant	CT vs. TT CC vs. TT	0.551 (0.191–1.586) –	0.296 –	128.593
Dominant	CC + CT vs. TT	0.551 (0.191–1.586)	0.296	128.593
Overdominant	CT vs. TT + CC	0.551 (0.191–1.586)	0.296	128.593
Recessive	CC vs. CT + TT	–	–	–
Additive	C	0.551 (0.191–1.586)	0.296	128.593
*TAS2R16* rs978739				
Codominant	CT vs. TT CC vs. TT	0.472 (0.170–1.315) 0.274 (0.070–1.068)	0.151 0.062	127.889
Dominant	CC + CT vs. TT	0.416 (0.159–1.088)	0.074	126.523
Overdominant	CT vs. TT + CC	0.642 (0.256–1.606)	0.343	129.020
Recessive	CC vs. CT + TT	0.397 (0.116–1.358)	0.141	127.992
Additive	C	0.514 (0.269–0.983)	**0.044**	125.934
*TAS2R16* rs1357949				
Codominant	AG vs. AA GG vs. AA	4.129 (1.321–12.898) –	**0.015**	120.846
Dominant	GG + AG vs. AA	4.979 (1.600–15.488)	**0.006**	120.305
Overdominant	AG vs. AA + GG	3.440 (1.106–10.705)	**0.033**	122.444
Recessive	GG vs. AG + AA	–	–	–
Additive	G	4.570 (1.553–13.447)	**0.006**	119.205

OR – odds ratio, AIC – Akaike information criteria; the underlined AIC value indicates the best genetic model; CI – confidence interval; *p*-value – significance level; Bonferroni corrected significance level when *p* = 0.05/3. The bolded results indicate significant differences between the groups

**Fig. 7:**
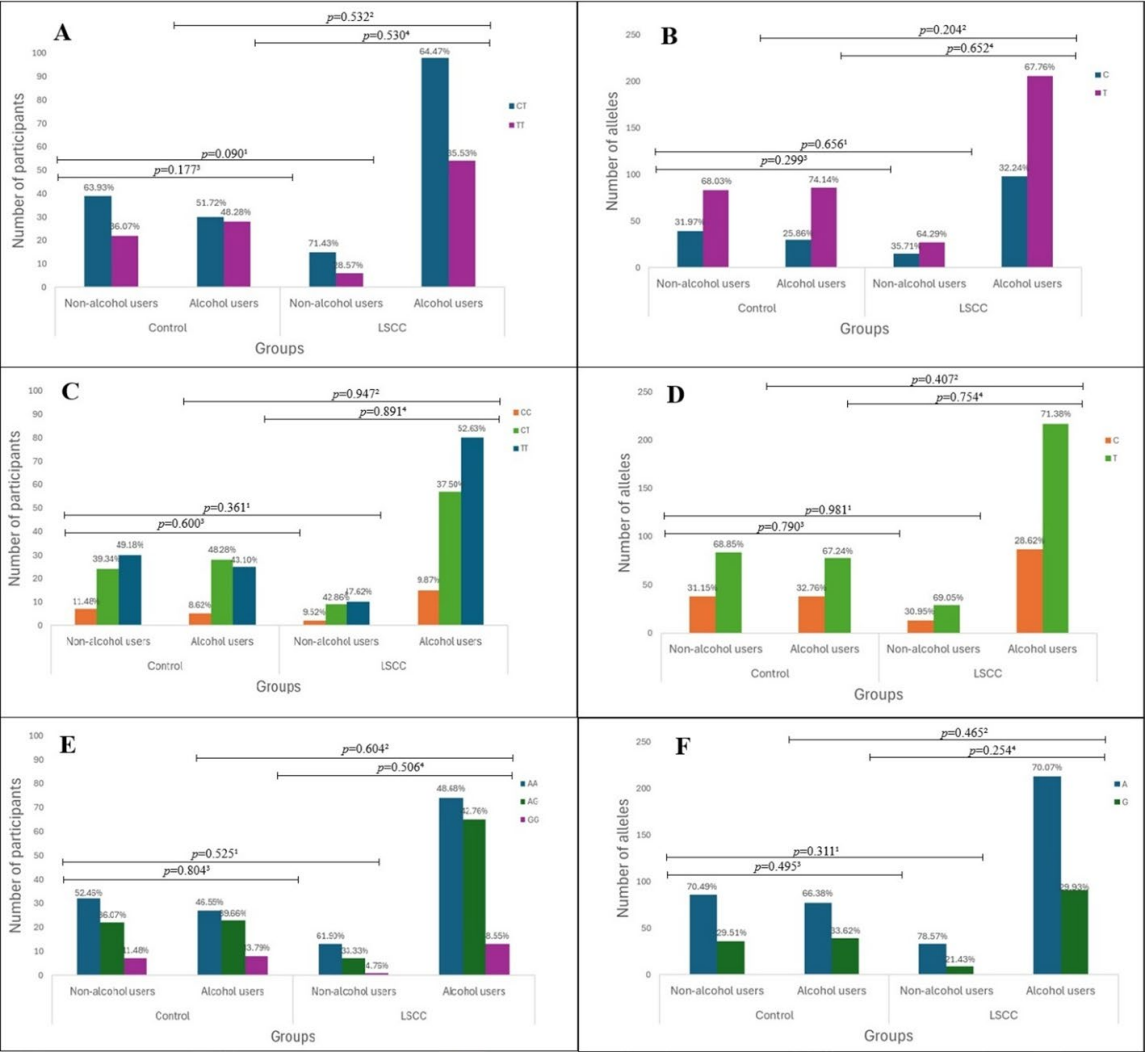
Genotype and allele frequencies of *TAS2R16* (rs860170, rs978739, rs1357949) in LSCC patients and controls according to alcohol consumption. A – genotype distribution of *TAS2R16* rs860170; B – allele distribution of *TAS2R16* rs860170; C – genotype distribution of *TAS2R16* rs978739; D – allele distribution of *TAS2R16* rs978739; E – genotype distribution of *TAS2R16* rs1357949; F – allele distribution of *TAS2R16* rs1357949; LSCC – laryngeal squamous cell carcinoma; *p*-value – significance level and Bonferroni corrected significance level when *p* = 0.05/3; *p*-value^1^ – control group non-alcohol users vs. LSCC group non-alcohol users; *p*-value^2^ – control group alcohol users vs. LSCC group alcohol users; *p*-value^3^ - control group non-alcohol users vs. alcohol users; *p*-value^4^ – LSCC group non-alcohol users vs. alcohol users

According to the alcohol consumption of the study subjects, all participants were divided into two groups: non-alcohol users and alcohol users. A comparison of genotype and allele distribution between alcohol drinkers in the control group and alcohol users in the LSCC group showed no statistically significant differences (Fig. 7).

Binomial logistic regression analysis of the rs860170, rs978739, and rs1357949 SNPs in LSCC patients who do not consume alcohol and in control subjects who do not use alcohol did not reveal any statistically significant results (Table 17).

**Table 17:** Binomial logistic regression analysis of *TAS2R16* (rs860170, rs978739, rs1357949) in non-alcohol users LSCC patients and non-drinking alcohol controls

Model	Genotype/allele	OR (95% CI)	*p*–value	AIC
*TAS2R16* rs860170				
Codominant	CT vs. TT CC vs. TT	1.410 (0.478–4.159) –	0.533 –	96.907
Dominant	CC + CT vs. TT	1.410 (0.478–4.159)	0.533	94.907
Overdominant	CT vs. TT + CC	1.410 (0.478–4.159)	0.533	94.907
Recessive	CC vs. CT + TT	–	–	–
Additive	C	1.410 (0.478–4.159)	0.533	94.907
*TAS2R16* rs978739				
Codominant	CT vs. TT CC vs. TT	1.125 (0.394–3.210) 0.857 (0.152–4.819)	0.826 0.861	97.194
Dominant	CC + CT vs. TT	1.065 (0.395–2.872)	0.902	95.290
Overdominant	CT vs. TT + CC	1.156 (0.423–3.160)	0.777	95.226
Recessive	CC vs. CT + TT	0.812 (0.155–4.254)	0.805	95.243
Additive	C	0.991 (0.475–2.070)	0.982	95.305
*TAS2R16* rs1357949				
Codominant	AG vs. AA GG vs. AA	0.783 (0.269–2.277) 0.352 (0.039–3.149)	0.654 0.350	96.187
Dominant	GG + AG vs. AA	0.679 (0.246–1.872)	0.454	94.738
Overdominant	AG vs. AA + GG	0.886 (0.311–2.525)	0.821	95.254
Recessive	GG vs. AG + AA	0.386 (0.045–3.335)	0.387	94.390
Additive	G	0.678 (0.305–1.508)	0.340	94.347

OR – odds ratio, AIC – Akaike information criteria; the underlined AIC value indicates the best genetic model; CI – confidence interval; *p*-value – significance level; Bonferroni corrected significance level when *p* = 0.05/3

However, we performed a logistic regression analysis of the selected SNPs in alcohol-using LSCC patients and alcohol-consuming controls but did not find statistically significant results (Table 18).

**Table 18:** Binomial logistic regression analysis of *TAS2R16* (rs860170, rs978739, rs1357949) in alcohol users, LSCC patients, and drinking alcohol controls

Model	Genotype/allele	OR (95% CI)	*p*–value	AIC
*TAS2R16* rs860170				
Codominant	CT vs. TT CC vs. TT	1.694 (0.918–3.126) –	0.092 –	246.683
Dominant	CC + CT vs. TT	1.694 (0.918–3.126)	0.092	246.683
Overdominant	CT vs. TT + CC	1.694 (0.918–3.126)	0.092	246.683
Recessive	CC vs. CT + TT	–	–	–
Additive	C	1.694 (0.918–3.126)	0.092	246.683
*TAS2R16* rs978739				
Codominant	CT vs. TT CC vs. TT	0.636 (0.336–1.203) 0.938 (0.310–2.837)	0.164 0.909	249.496
Dominant	CC + CT vs. TT	0.682 (0.371–1.254)	0.218	247.986
Overdominant	CT vs. TT + CC	0.643 (0.349–1.184)	0.156	247.509
Recessive	CC vs. CT + TT	1.161 (0.402–3.352)	0.783	249.437
Additive	C	0.828 (0.526–1.304)	0.415	248.854
*TAS2R16* rs1357949				
Codominant	AG vs. AA GG vs. AA	1.031 (0.539–1.972) 0.593 (0.221–1.587)	0.926 0.298	250.296
Dominant	GG + AG vs. AA	0.918 (0.501–1.683)	0.782	249.438
Overdominant	AG vs. AA + GG	1.137 (0.614–2.106)	0.683	249.347
Recessive	GG vs. AG + AA	0.585 (0.229–1.494)	0.262	248.305
Additive	G	0.846 (0.538–1.331)	0.470	248.995

OR – odds ratio, AIC – Akaike information criteria; the underlined AIC value indicates the best genetic model; CI – confidence interval; *p*-value – significance level; Bonferroni corrected significance level when *p* = 0.05/3

Moreover, a logistic regression analysis of the selected SNPs was also performed for non-alcohol-using and alcohol-consuming controls and non-alcohol-using and alcohol-consuming LSCC patients, although statistically significant results were not found (Tables 19 and 20).

**Table 19:** Binomial logistic regression analysis of *TAS2R16* (rs860170, rs978739, rs1357949) in non-alcohol users and drinking alcohol controls

Model	Genotype/allele	OR (95% CI)	*p*–value	AIC
*TAS2R16* rs860170				
Codominant	CT vs. TT CC vs. TT	0.604 (0.290–1.259) –	0.179 –	165.070
Dominant	CC + CT vs. TT	0.604 (0.290–1.259)	0.179	165.070
Overdominant	CT vs. TT + CC	0.604 (0.290–1.259)	0.179	165.070
Recessive	CC vs. CT + TT	–	–	–
Additive	C	0.604 (0.290–1.259)	0.179	165.070
*TAS2R16* rs978739				
Codominant	CT vs. TT CC vs. TT	1.400 (0.654–2.996) 0.857 (0.242–3.035)	0.386 0.811	167.871
Dominant	CC + CT vs. TT	1.277 (0.620–2.631)	0.507	166.451
Overdominant	CT vs. TT + CC	1.439 (0.695–2.987)	0.327	165.928
Recessive	CC vs. CT + TT	0.728 (0.217–2.437)	0.606	166.625
Additive	C	1.077 (0.624–1.861)	0.789	166.822
*TAS2R16* rs1357949				
Codominant	AG vs. AA GG vs. AA	1.239 (0.570–2.695) 1.354 (0.435–4.220)	0.589 0.601	168.456
Dominant	GG + AG vs. AA	1.267 (0.617–2.603)	0.520	166.478
Overdominant	AG vs. AA + GG	1.165 (0.555–2.445)	0.687	166.730
Recessive	GG vs. AG + AA	1.234 (0.417–3.652)	0.704	166.748
Additive	G	1.185 (0.706–1.989)	0.520	166.479

OR – odds ratio, AIC – Akaike information criteria; the underlined AIC value indicates the best genetic model; CI – confidence interval; *p*-value – significance level; Bonferroni corrected significance level when *p* = 0.05/3

**Table 20:** Binomial logistic regression analysis of *TAS2R16* (rs860170, rs978739, rs1357949) in non-alcohol users and drinking alcohol LSCC patients

Model	Genotype/allele	OR (95% CI)	*p*–value	AIC
*TAS2R16* rs860170				
Codominant	CT vs. TT CC vs. TT	0.726 (0.266–1.980) –	0.532 –	129.504
Dominant	CC + CT vs. TT	0.726 (0.266–1.980)	0.532	129.504
Overdominant	CT vs. TT + CC	0.726 (0.266–1.980)	0.532	129.504
Recessive	CC vs. CT + TT	–	–	–
Additive	C	0.726 (0.266–1.980)	0.532	129.504
*TAS2R16* rs978739				
Codominant	CT vs. TT CC vs. TT	0.792 (0.302–2.073) 0.938 (0.186–4.715)	0.634 0.938	131.681
Dominant	CC + CT vs. TT	0.818 (0.328–2.040)	0.667	129.724
Overdominant	CT vs. TT + CC	0.800 (0.317–2.017)	0.636	129.688
Recessive	CC vs. CT + TT	1.040 (0.220–4.908)	0.960	129.907
Additive	C	0.901 (0.459–1.771)	0.763	129.819
*TAS2R16* rs1357949				
Codominant	AG vs. AA GG vs. AA	1.631 (0.614–4.335) 2.284 (0.275–18.982)	0.326 0.445	130.509
Dominant	GG + AG vs. AA	1.713 (0.671–4.369)	0.260	128.608
Overdominant	AG vs. AA + GG	1.494 (0.571–3.912)	0.413	129.220
Recessive	GG vs. AG + AA	1.871 (0.232–15.082)	0.557	129.503
Additive	G	1.577 (0.720–3.457)	0.255	128.522

OR – odds ratio, AIC – Akaike information criteria; the underlined AIC value indicates the best genetic model; CI – confidence interval; *p*-value – significance level; Bonferroni corrected significance level when *p* = 0.05/3

### TAS2R16 serum concentrations‘ association with LSCC

**Fig. 8:**
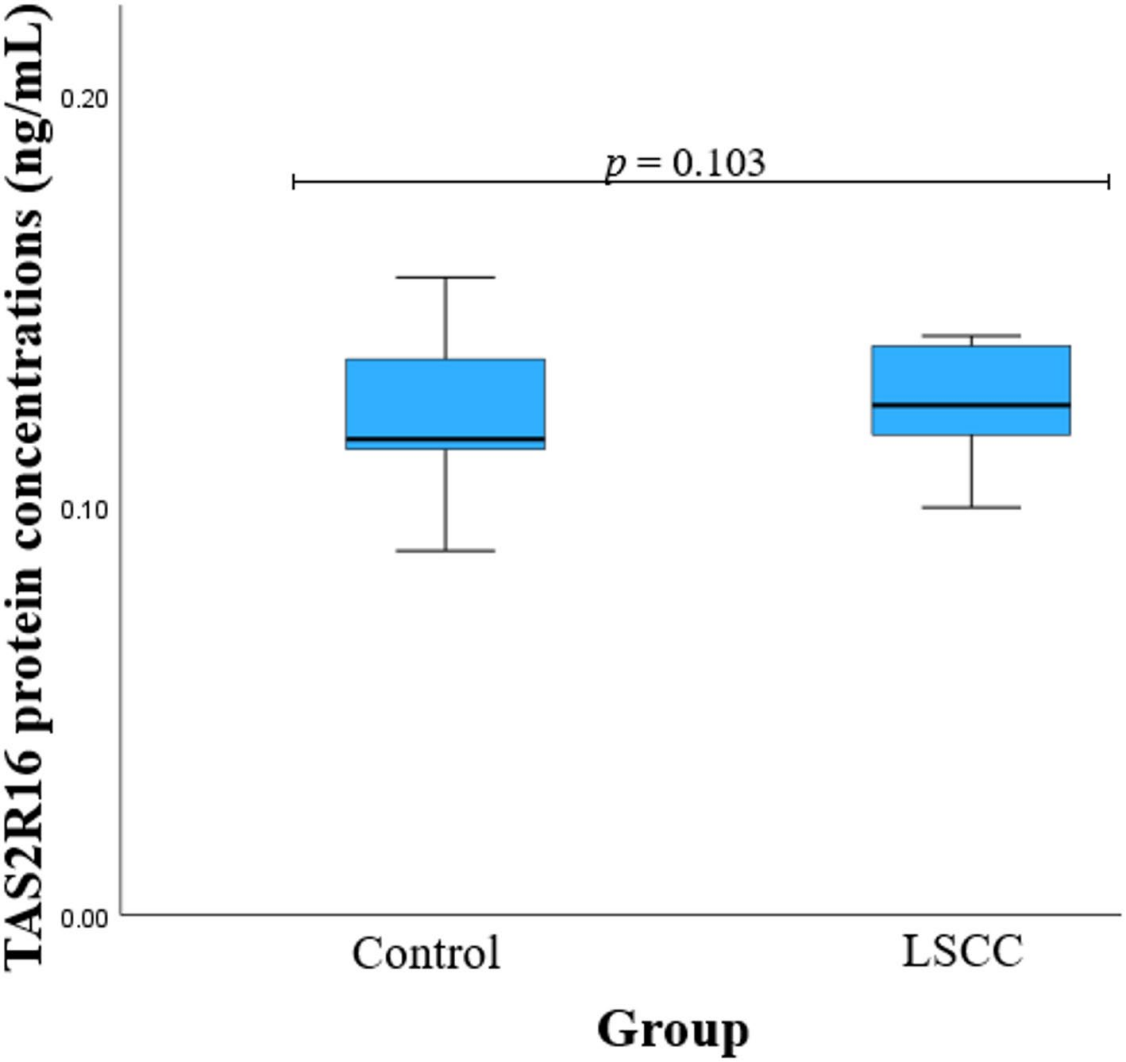
TAS2R16 protein concentrations in control and LSCC groups

Protein TAS2R16 concentrations were measured in groups of patients with LSCC and healthy subjects. We found that TAS2R16 serum levels showed no statistically significant difference between LSCC patients and control group subjects (0.124 (0.024) ng/mL vs. 0.116 (0.027) ng/mL, *p* = 0.103) (Fig. 8).

A TAS2R16 concentration comparison between study groups and genotypes was performed and showed statistically significant differences between the two groups. We found that *TAS2R16* rs978739 CT carriers had higher TAS2R16 serum levels in the LSCC than the control group (0.129 (0.054) vs. 0.115 (0.017); *p* = 0.027). A Mann–Whitney U test was used to compare TAS2R16 concentrations between the two groups. The bars represent the median with the interquartile range (Table 21).

**Table 21:** Genotype distribution of SNPs and TAS2R16 concentration levels between patients with LSCC and the control group

Genotype	TAS2R16 concentration		*p*–value
	Control Median (IQR)	LSCC Median (IQR)	
*TAS2R16* rs860170			
TT	0.115 (0.016)	0.124 (0.016)	0.180
CT	0.118 (0.030)	0.127 (0.042)	0.427
CC	–	–	–
*TAS2R16* rs978739			
TT	0.116 (0.721)	0.118 (0.017)	0.661
CT	0.115 (0.017)	0.129 (0.054)	**0.027**
CC	0.118 (1.126)	0.149 (–)	0.439
*TAS2R16* rs1357949			
AA	0.151 (2.208)	0.128 (0.058)	0.937
AG	0.116 (0.011)	0.118 (0.014)	0.356
GG	–	–	–

LSCC – laryngeal squamous cell carcinoma; *p*-value – significance level and Bonferroni corrected significance level when *p* = 0.05; the bolded results indicate significant differences between the groups

### Haplotype analysis of *TAS2R16* rs860170, rs978739, and rs1357949

A high pairwise linkage disequilibrium (LD) was observed between the polymorphisms *TAS2R16* rs978739 and rs1357949, rs860170 and rs978739, rs860170 and rs1357949 (Table 22).

**Table 22:** Linkage disequilibrium between *TAS2R16* SNPs

SNPs	D’	*r* ^2^
rs860170-rs978739	0.889	0.175
rs860170-rs1357949	0.869	0.154
rs978739- rs1357949	0.913	0.176

SNPs – single nucleotide polymorphisms; *D’* is the deviation between the expected haplotype frequency and the observed frequency [*D*’ scale: 0.1]; *r*^2^ is the squared correlation coefficient of the haplotype frequencies [*r*^2^ scale: 0.1]

A haplotype association analysis was performed of *TAS2R16* rs860170, rs978739, and rs1357949 in patients with LSCC compared with a control group. Statistical analysis showed no statistically significant associations (Table 23).

**Table 23:** Haplotype association of *TAS2R16* (rs860170, rs978739, rs1357949) with the predisposition to LSCC occurrence

Haplotype	*TAS2R16* rs860170	*TAS2R16* rs978739	*TAS2R16* rs1357949	Frequency (%)		OR (95% CI)	*p*–value
				Control	LSCC		
1	T	C	A	0.306	0.304	1.00	–
2	C	T	A	0.277	0.316	1.20 (0.84–1.70)	0.32
3	T	T	G	0.298	0.278	0.93 (0.70–1.25)	0.64
4	T	T	A	0.074	0.089	1.12 (0.72–1.74)	0.62
5	C	T	G	0.017	0.003	0.13 (0.02–1.02)	0.052
Rare	*	*	*	–	–	0.44 (0.16–1.19)	0.1

LSCC – laryngeal squamous cell carcinoma; OR – odds ratio; CI – confidence interval; *p*-value – significance level; *All haplotypes with less than 1% frequencies were grouped and labeled as “rare” haplotypes

### Association between *TAS2R16* rs860170, rs978739, and rs1357949 and five-year survival rate

**Fig. 9:**
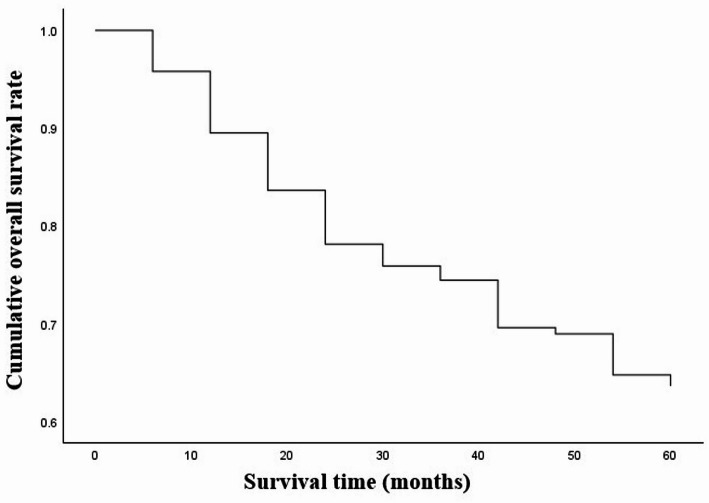
The five-year overall survival rate of the patients included in the LSCC group

The five-year overall survival (OS) rate of selected 238 LSCC patients including all causes of death was 51% (Fig. 9).

**Fig. 10:**
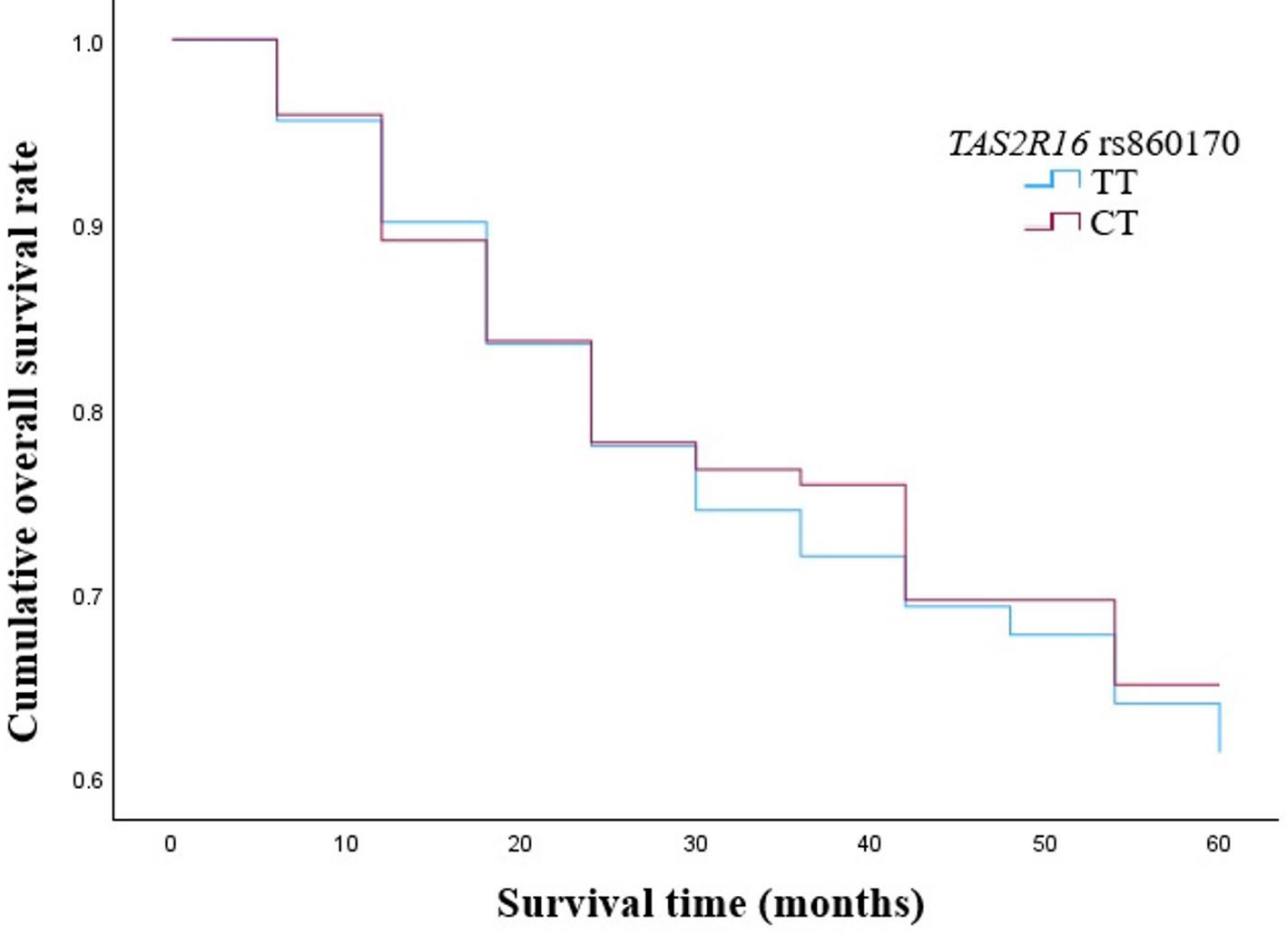
Five-year survival rate according to the distribution of *TAS2R16* rs860170 genotypes

However, we analyzed LSCC patients’ five-year survival rate and the genotype distributions of *TAS2R16* rs860170, rs978739, and rs1357949. We did not find any statistically significant differences between the genotypic distribution of *TAS2R16* rs860170 and the survival rate of the LSCC patients (*p* = 0.794) (Fig. 10).

**Fig. 11:**
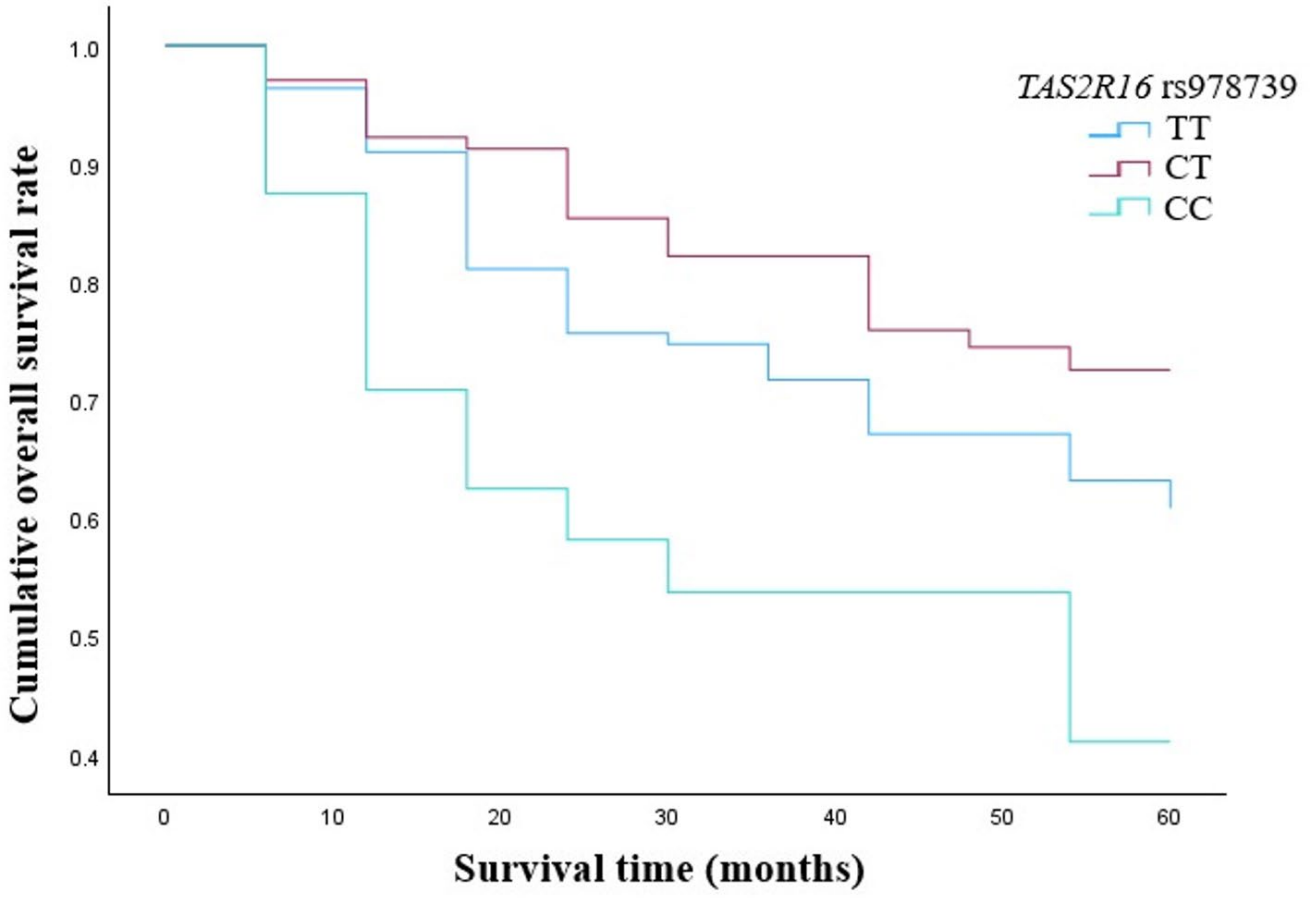
Five-year survival rate according to the distribution of *TAS2R16* rs978739 genotypes

Analyzing LSCC patients’ five-year survival rate and the genotype distribution of *TAS2R16* rs978739 we clarified that subjects carrying CC genotype had a statistically significantly poorer five-year survival rate (five-year survival 25%) than those carrying TT and CT genotypes (five-year survival 56%, *p* = 0.043 and 53%, *p* = 0.002, respectively) (Fig. 11).

**Fig. 12:**
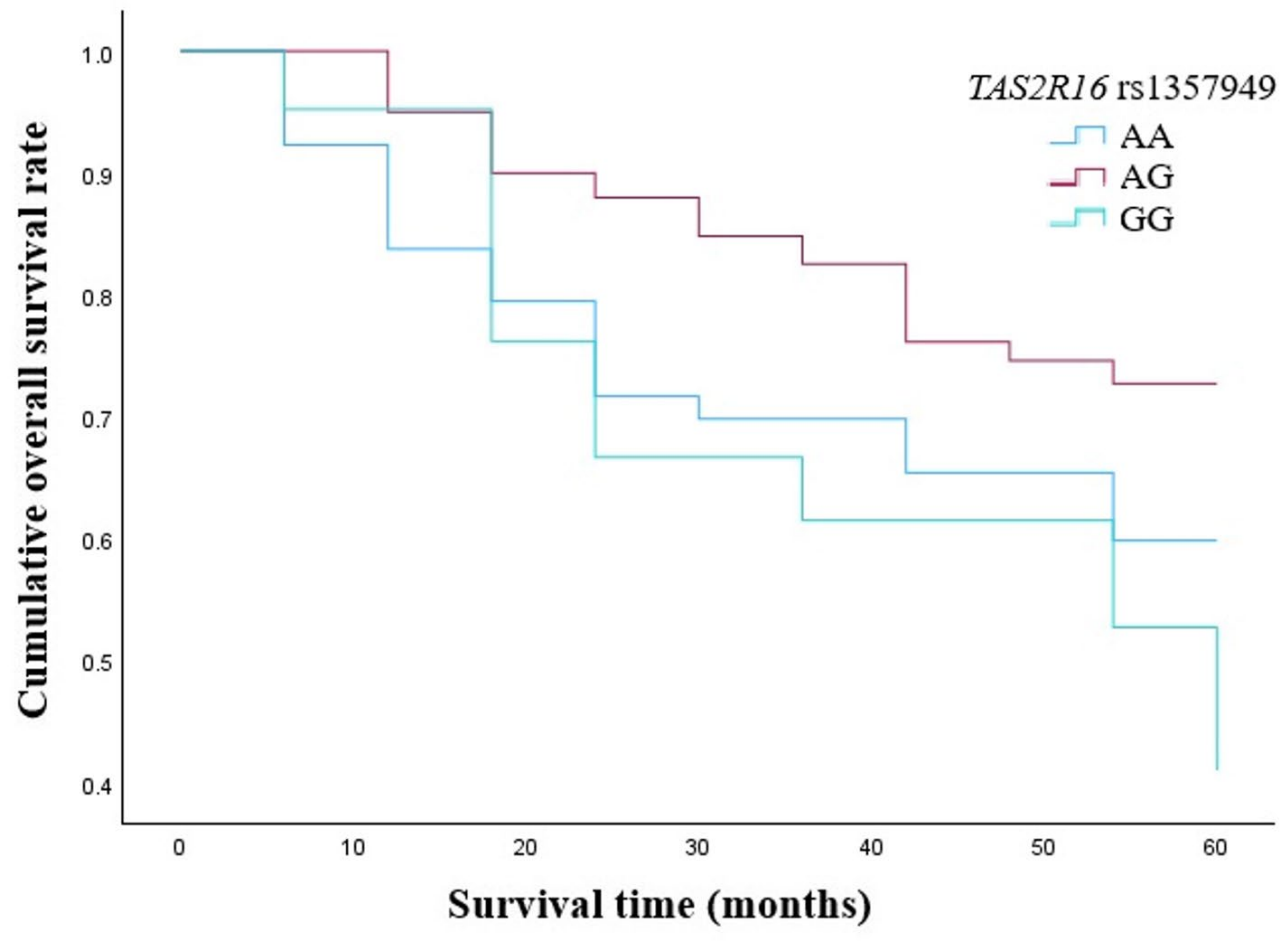
Five-year survival rate according to the distribution of *TAS2R16* rs1357949 genotypes

Results revealed five-year survival based on the distribution of the genotypes of the *TAS2R16* rs1357949 that the carriers of AG genotype had a statistically significantly greater five-year survival rate (five-year survival 67%) than those carrying AA and GG genotypes (five-year survival 40%, *p* = 0.013 and 41%, *p* = 0.048, respectively) (Fig. 12).

## Discussion

During this study, we investigated the possible association between *TAS2R16* SNPs and TAS2R16 protein serum levels in patients with laryngeal squamous cell carcinoma in Lithuania. We performed genotyping analysis of three specific *TAS2R16* SNPs: rs860170, rs978739, and rs1357949. The analysis was performed on two groups consisting of 312 patients with LSCC and 320 control subjects. For the first time, we examined the serum levels of *TAS2R16* rs860170, rs978739, and rs1357949 in LSCC patients. Although studies have examined the impact of the *TAS2R16* rs860170, rs978739, and rs1357949 polymorphisms examined in this work on other diseases, whose development may be linked to autoimmune diseases, inflammation, and other factors [13, 15]. At the same time, there is no information in the scientific literature regarding the association between these polymorphisms and LSCC. The fundamental cause of TAS2R16 link to illnesses is that food decisions are influenced by the altered sensitivity to bitter taste brought on by desire and reluctance. Changes in desire and reluctance brought on by a heightened sensitivity to bitter taste also influence dietary choices, which is the fundamental cause of its relationship with LSCC. Furthermore, forming dietary habits may have an impact on nutritional status later and raise the risk of chronic illnesses and long-term health issues.

The gene encoding *TAS2R16* is located on the long arm of chromosome 7, at position 31 (7q31.32) and is 997 bp long [22]. The bitter taste receptor family’s *TAS2R16* gene, sensitive to substances including salicin, amygdalin, and sinigrin, has recently been subjected to selection pressure [17]. These substances, present in many foods, have pharmacological actions including anti-inflammatory and cancer-inhibiting qualities [23–25]. Pears and wheat have arbutin, which suppresses bladder cancer [25]; fruit seeds contain amygdalin, which may influence cancer cells [26]; Brassicaceae plants include sinigrin, which may protect against colorectal cancer [27]; and willow bark has salicin, which functions similarly to aspirin [28]. According to research, the TAS2R16 receptor plays a part in identifying advantageous chemicals the organism interacts with. The effectiveness of the different substances may be impacted by a receptor’s reduced function, which might eventually put the organism at a disadvantage.

Beyond bitter taste receptor’s role in bitter taste perception, innate immunity, thyroid function, heart physiology, and other biological functions are all impacted by TAS2Rs [29–31]. Moreover, TAS2Rs have been investigated in head and neck squamous cell carcinoma (HNSCC), breast, and ovarian malignancies [32–34]. Studies demonstrate that some bitter agonists that activate different TAS2Rs raise nuclear and mitochondrial Ca2+, which causes squamous airway epithelium and HNSCC cells to undergo apoptosis [34, 35]. Furthermore, in the study by Mirza et al., patients with head and neck cancer showed anomalies in taste, and based on therapy, it was proposed that radiation exposure was the origin of these deficiencies [36].

It has been proposed that polymorphic variants in *TAS2R16* affect the perceptions, preferences, or consumption of common beverages that contain phytochemicals and other pharmacologically active ingredients associated with chronic diseases [16]. These variations also confer differential response in vitro through functional alterations in the receptor [37]. Genetic polymorphisms and environmental factors can influence the TAS2R16 receptor’s ability to recognize harmful and beneficial substances, potentially affecting lifespan. Recent studies suggest taste receptors like TAS2R16 also have non-gustatory roles, such as influencing respiratory function in response to harmful stimuli, which may contribute to healthy aging [38].

HNSCC, including LSCC, is influenced by various genetic alterations beyond *TAS2R16*. Several well-established genes play a critical role in tumorigenesis, and their interaction with clinical features is widely studied. *SPINK5*, serine peptidase inhibitor Kazal type 5, also known as lympho-epithelial Kazal-type-related inhibitor, is a member of the serine protease inhibitor Kazal type family, is located in the 5q32 region of the chromosome and contains 15 potential inhibitory domains [39]. Prior research revealed that, in comparison to the matched neighboring normal tissues, SPINK5 was downregulated 9.7 times in 22 head and neck squamous cell carcinoma tissues [40]. The same team’s subsequent research showed that downregulated SPINK5 facilitated the invasion, colony formation, and proliferation of HNSCC cells [41]. Furthermore, it was shown that oral squamous cell carcinoma had decreased levels of LEKTI, a big protein encoded by the *SPINK5* gene, with a shorter OS being associated with a higher KLK5/SPINK5 mRNA ratio [42]. Liu and co-authors found that SPINK5 was downregulated in tumor tissues compared to normal tissues, showing a significant correlation with LSCC. Their findings demonstrated a correlation between *SPINK5* and the DNA damage and repair pathways as well as the carcinogenesis pathways. Low *SPINK5* expression in Liu et al. study was associated with worse survival and worse outcomes for LSCC patients. It’s probable that *SPINK5* contributes to a bad prognosis for LSCC patients by interfering with various signaling pathways [43]. However, RNA transcripts longer than 200 nucleotides are known as long non-coding RNAs, or lncRNAs. They have an important role in the control of gene expression, chromatin remodeling, splicing, and intracellular trafficking, but they do not have the functional duty of encoding proteins [44]. Many different kinds of lncRNAs have been shown to be useful prognostic biomarkers and therapeutic targets for LSCC or HNSCC as a result of current research examining the modalities of cell death in these cancers. Nevertheless, little is known about how disulfidptosis-related lnRNAs (DRlncRNAs) contribute to laryngeal squamous cell carcinoma (LSCC) [4]. The use of the DRlncRNAs signature in a number of malignant tumors, such as liver hepatocellular carcinoma [45], lung adenocarcinoma [46], and colon adenocarcinoma [47], has been well documented in earlier research, demonstrating its important prognostic and therapeutic utility. The study done by Zhang et al. aimed to determine the prognostic DRlncRNAs in LSCC, which can offer critical insights into the underlying signaling pathways and mechanisms involved. Significant differences emerged between the high-risk and low-risk groups of the LSCC patients, particularly in immune-related processes and metabolic dysfunction. The high-risk group exhibited activation of metabolic pathways (glutathione metabolism, pentose phosphate pathway) and showed higher levels of activated mast cells and eosinophils but lower activated NK cell levels, indicating a potentially compromised anti-tumor immune response. In contrast, the low-risk group displayed immune-related pathway activation (IgA production, autoimmune diseases) and had greater immune cell infiltration, as confirmed by a significantly higher immune score. *In vitro* experiments revealed distinct behaviors of DRlncRNAs, highlighting their potential role in tumor progression and immune regulation. Ultimately, the DRlncRNAs signature presents a robust biomarker capable of predicting both prognosis and therapeutic responses in LSCC patients [4].Notably, *TAS2R16*, a bitter taste receptor gene, has emerged as another significant genetic factor in LSCC. Discussing the results of our research, we found that the distribution of TT, CT, and CC genotypes of the *TAS2R16* rs860170 was statistically significantly different in LSCC patients compared to the control group (*p* = 0.002). Barontini and co-authors analyzed *TAS2R16* rs860170 influence on colorectal cancer patients, although, they did not find any statistically significant associations [48]. Another study investigated *TAS2R16* SNPs’ association with benign tumour – pituitary adenoma (PA). A study showed no statistically significant correlation between rs860170 and PA development [49]. Gedvilaite et al. studied the influence of *TAS2R16* gene variants on the development of chronic disease. They found that the CC genotype and C allele were statistically significantly more frequent in the multiple sclerosis patients than in the healthy individuals (*p* < 0.001 and *p* = 0.008, respectively) [50].

We also examined the impact of selected SNPs on the clinical features of the LSCC. Results showed that the distribution of TT, CT, and CC genotypes of *TAS2R16* rs860170 is statistically significantly different in both early-stage and late-stage LSCC patients compared to the control group (*p* = 0.035 and *p* = 0.023, respectively). We determined that *TAS2R16* rs860170 CT genotype is associated with 1.5-fold increase odds of developing late-stage LSCC (*p* = 0.043) Also, a statistically significant difference was observed in the distribution of TT, CT, and CC genotypes of *TAS2R16* rs860170 between LSCC patients without metastasis and the control group (*p* = 0.005) and between patients with poorly differentiated LSCC and the control group (*p* = 0.011). While other scientists analyzed *TAS2R16* SNPs’ influence on clinical features of PA, they found that the CT genotype of the rs860170 reduces the likelihood of developing non-invasive PA by 1.9-fold under the codominant and overdominant model (*p* = 0.024 and *p* = 0.030, respectively). Nevertheless, under the dominant model, rs860170 CT + CC genotypes reduce the odds of developing non-invasive PA by two times (*p* = 0.021), and under the additive model, each C allele reduces these odds by two times (*p* = 0.018) [49]. In another study done by Liu and co-authors tumour features, such as advanced T, advanced N, and advanced M stages, were linked to an increased risk of early mortality from all causes and from cancer specifically [51].

According to earlier research, smoking is a risk factor in and of itself for a lower specific survival rate in LSCC [51]. Considering that smoking and alcohol consumption are risk factors for head and neck cancer [10], we suggested that these lifestyle habits may have an important role in the development of LSCC and should be further investigated. The analysis of genotype and allele distribution revealed that the distribution of the *TAS2R16* rs860170 TT, CT, and CC genotypes was statistically significantly different between smoking controls and LSCC patients who smoked (*p* = 0.040), suggesting a potential role of this variant in LSCC among smokers. *TAS2R16* rs1357949 AA, AG, and GG genotypes were statistically significantly different between non-smoking controls and non-smoking LSCC patients (*p* = 0.029). Also, the distribution of these genotypes is statistically significantly different between non-smokers of the LSCC group and LSCC smokers (*p* = 0.009). Our findings show that the *TAS2R16* rs1357949 polymorphism is significantly associated with LSCC in non-smokers, as its genotype distribution differs between non-smoking controls and non-smoking LSCC patients. A notable difference in rs1357949 genotype distribution is also observed between non-smoking LSCC patients and smoking LSCC patients, indicating that this SNP may influence LSCC risk differently in smokers and non-smokers. The *TAS2R16* rs1357949 G allele is less common in non-smokers of the LSCC group than in the non-smokers of the control group (*p* = 0.006), and the smokers than in non-smokers when comparing only LSCC patients (*p* = 0.003), proposing a potential protective effect of this allele in non-smokers. *TAS2R16* rs978739 C allele is more frequent in non-smokers than in smokers of the LSCC group (*p* = 0.033), further indicating that genetic susceptibility to LSCC may differ by smoking status. The binomial logistic regression analysis reveals significant associations between *TAS2R16* polymorphisms and both LSCC development and smoking behavior, highlighting potential genetic risk factors and protective effects. This analysis in smokers with LSCC patients and smoking controls showed that the *TAS2R16* rs860170 CT genotype was found to increase the odds of developing LSCC in smokers by 2.5 times under the codominant and overdominant models, while the CT + CC models increase these odds by 2.5-fold under the dominant model, also each C allele increases these odds by 2.5 times (*p* = 0.044 in all models). Our results imply that rs860170 of the *TAS2R16* may act as a risk factor for LSCC in smokers, increasing susceptibility to the disease. The results showed that *TAS2R16* rs1357949 GG and AG genotypes together are associated with 4.2-fold decreased odds of developing LSCC in non-smoking patients under the dominant model (*p* = 0.016). Also, each rs1357949 G allele decreased the odds of LSCC occurrence in non-smokers by 3.8-fold under the additive model (*p* = 0.014), suggesting a potential protective effect. Analysis revealed that *TAS2R16* rs978739 each C allele is associated with reduced odds of smoking for LSCC patients by 1.9-fold under the additive model (*p* = 0.044), indicating a possible protective role against smoking. Moreover, *TAS2R16* rs1357949 AG genotype was found to increase the odds of smoking for LSCC patients by 4.2-fold and 3.4-fold under the codominant and overdominant models (*p* = 0.015 and *p* = 0.033, respectively), while GG and AG genotypes together increase these odds by 5 times under the dominant model (*p* = 0.006). Also, each of the rs1357949 G alleles increases the odds of the smoking habit for LSCC patients by 4.6-fold under the additive model (*p* = 0.006), suggesting a genetic influence on smoking behavior. So far, only other *TAS2R16* genetic variants appear to influence alcohol and nicotine dependence [12, 19]. Since this is the first study to examine the associations between *TAS2R16* (rs860170, rs978739, and rs1357949) polymorphisms and human habits such as smoking and alcohol drinking, our findings provide new insights into the potential role of this receptor in lifestyle factors and LSCC risk, warranting further investigation in future studies. Our study implies that *TAS2R16* polymorphisms may contribute to LSCC susceptibility, with distinct effects in smokers and non-smokers, specifically the rs1357949 G allele may have a protective role, particularly in non-smokers. Our results imply that the C allele of *TAS2R16* rs860170 may be associated with an increased risk under multiple inheritance models. However, *TAS2R16* rs1357949 G allele appears to have a protective effect against LSCC in non-smokers, but it is associated with a significantly higher likelihood of smoking among LSCC patients. Also, *TAS2R16* rs978739 C allele may be protective against smoking in LSCC patients, which could have implications for smoking cessation strategies. These findings suggest a strong gene-environment interaction between *TAS2R16* SNPs and smoking in LSCC risk. Further research is needed to explore these genetic influences on smoking behavior and cancer susceptibility.

TAS2R16 protein concentrations were also measured in the patients’ and controls’ serum. TAS2R16 concentration comparison between study groups and genotypes was performed. We determined that the TAS2R16 serum levels in the LSCC were greater in *TAS2R16* rs978739 CT genotype carriers than in the control group (*p* = 0.027). Different genotypes of the *TAS2R16 (*rs860170, rs978739, and rs1357949) were also compared in terms of TAS2R16 serum levels in patients with PA and controls. PA patients with the TT or CT genotype of the *TAS2R16* rs860170 had higher protein levels than the healthy control group (*p* = 0.031 and *p* = 0.006, respectively). Serum levels of TAS2R16 were greater in PA patients with the TT or CT genotype than in healthy individuals, according to *TAS2R16* rs978739 SNV (*p* = 0.025 and *p* = 0.019, respectively). Furthermore, PA patients with the AA or AG genotype of the *TAS2R16* rs1357949 SNV had higher TAS2R16 protein concentrations than did healthy individuals (*p* = 0.005 and *p* = 0.007, respectively) [49]. The differences in TAS2R16 levels based on genotype and condition indicate that these genetic variations may play a role in the pathophysiology of LSCC and PA, possibly affecting disease susceptibility or progression.

In our study we examined the the five-year overall survival (OS) rate of LSCC patients according to the selected *TAS2R16* SNPs. We determined that patients who have the CC genotype of the rs978739 had a statistically significantly poorer five-year survival rate than those carrying TT and CT genotypes (*p* = 0.043 and *p* = 0.002, respectively). According to rs1357949, the five-year survival rate for carriers of the AG genotype was statistically substantially higher than for those carrying the AA and GG genotypes (*p* = 0.013 and *p* = 0.048, respectively). Pasvenskaite and co-authors found that the five-year overall survival rate of 300 LSCC patients was 65%. These scientists determined that AA genotype at *IL-9* rs1859430 is statistically significant association with poorer LSCC-specific five-year survival rate [52]. In other study done by Liu et al. the 3-month mortality rates among patients with newly diagnosed supraglottic LSCC were calculated. Scientist found that 8.38% of the supraglottic LSCC patients died within 3 months following diagnosis [51].

The observed associations between *TAS2R16* genetic variants, lifestyle factors like smoking, and LSCC risk emphasize the need to further explore these interactions. Our findings open new avenues for research into the potential of TAS2R16 as a biomarker for LSCC and other related cancers, while also suggesting the importance of dietary habits and lifestyle choices in cancer prevention. Although there were notable discoveries in this study, we recognize that our work also has several future directions. For example, conducting studies in diverse populations will help validate the observed associations and ensure broader applicability of the findings. Further research is needed to elucidate the molecular mechanisms by which *TAS2R16* influences inflammation in LSCC and to clarify the exact effect of the protein on the occurrence and progression of the disease. Also, investigating additional bitter taste receptor genes may uncover their potential roles in cancer susceptibility and progression.

## Conclusion

This study represents the first investigation into the association between *TAS2R16* SNPs (rs860170, rs978739, and rs1357949) and TAS2R16 protein serum levels in patients with LSCC in Lithuania. Our findings reveal that specific *TAS2R16* genotypes are significantly associated with LSCC, suggesting a potential role of *TAS2R16* genetic polymorphisms in the disease’s development and clinical features. The study also demonstrates that the TAS2R16 protein serum levels are elevated in LSCC patients carrying *TAS2R16* rs978739 CT genotype, further supporting the involvement of this receptor in LSCC pathophysiology.

Future studies should aim to confirm these associations in larger, more diverse populations and investigate the underlying mechanisms of *TAS2R16*’s influence on cancer development, considering both genetic predispositions and environmental factors such as diet and lifestyle.

## Data Availability

The data presented in this study are available on request from the corresponding author.
